# Functional Characterization of the Cnidarian Antiviral Immune Response Reveals Ancestral Complexity

**DOI:** 10.1093/molbev/msab197

**Published:** 2021-06-28

**Authors:** Magda Lewandowska, Ton Sharoni, Yael Admoni, Reuven Aharoni, Yehu Moran

**Affiliations:** Department of Ecology, Evolution and Behavior, Alexander Silberman Institute of Life Sciences, Faculty of Science, Hebrew University of Jerusalem, Jerusalem, Israel

**Keywords:** evolution of immunity, innate immunity, RNA interference, Cnidaria, double-stranded RNA

## Abstract

Animals evolved a broad repertoire of innate immune sensors and downstream effector cascades for defense against RNA viruses. Yet, this system varies greatly among different bilaterian animals, masking its ancestral state. In this study, we aimed to characterize the antiviral immune response of the cnidarian *Nematostella vectensis* and decipher the function of the retinoic acid-inducible gene I (RIG-I)-like receptors (RLRs) known to detect viral double-stranded RNA (dsRNA) in bilaterians but activate different antiviral pathways in vertebrates and nematodes. We show that polyinosinic:polycytidylic acid (poly(I:C)), a mimic of long viral dsRNA and a primary ligand for the vertebrate RLR melanoma differentiation-associated protein 5 (MDA5), triggers a complex antiviral immune response bearing features distinctive for both vertebrate and invertebrate systems. Importantly, a well-characterized agonist of the vertebrate RIG-I receptor does not induce a significant transcriptomic response that bears signature of the antiviral immune response, which experimentally supports the results of a phylogenetic analysis indicating clustering of the two *N. vectensis* RLR paralogs (NveRLRa and NveRLRb) with MDA5. Furthermore, the results of affinity assays reveal that NveRLRb binds poly(I:C) and long dsRNA and its knockdown impairs the expression of putative downstream effector genes including RNA interference components. Our study provides for the first time the functional evidence for the conserved role of RLRs in initiating immune response to dsRNA that originated before the cnidarian–bilaterian split and lay a strong foundation for future research on the evolution of the immune responses to RNA viruses.

## Introduction

**Fig. 1.**
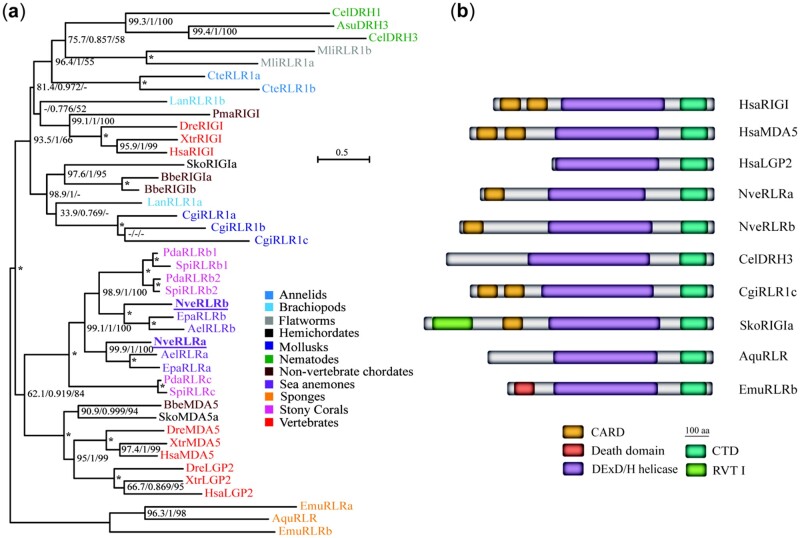
Phylogenetic relationship of metazoan RLRs. (*a*) Maximum likelihood consensus phylogenetic tree of representative RLR sequences, bootstrap values above 50% are presented for each node. Support values of the SH-aLRT (50–100), approximate Bayes test (0.6–1.0), and ultrafast bootstrap replicates (50–100) appear from left to right near each relevant node. A dash represents a lower value in the relevant test. Asterisks represent nodes where the three test values are perfect (i.e., 100/1.0/100). Ael, *Anthopleura elegantissima*; Aqu, *Amphimedon queenslandica*; Asu, *Ascaris suum*; Bbe, *Branchiostoma belcheri*; Cte, *Capitella teleta*; Cgi, *Crassostrea gigas*; Cel, *Caenorhabditis elegans*; Dre, *Danio rerio*; Emu, *Ephydatia muelleri*; Epa, *Exaiptasia pallida*; Hsa, *Homo sapiens*; Lan, *Lingula anatina*; Mli, *Macrostomum lignano*; Nve, *Nematostella vectensis*; Pda, *Pocillopora damicronis*; Pma, *Petromyzon marinus*; Sti, *Stylophora pistillata*; Xtr, *Xenopus tropicalis*. (*b*) Schematic representation of selected RLR representatives of major phylogenetic groups. CARD, caspase recruitment domain; CTD, C-terminal domain; RVT I, reverse transcriptase.

The immune system has long been known for its remarkable patterns of rapid evolution owing to strong selective drivers such as fast-evolving pathogens (Koonin and Dolja 2013; tenOever 2016). Thus, revealing conservation among phylogenetically distant lineages can provide unprecedented insights into the evolution of these defense mechanisms. For instance, it has been recently reported that both eukaryotic antiviral DNA-sensing mechanism driven by cGAS-STING axis and the downstream inhibitors of virus replication called viperins have originated in prokaryotes as anti-bacteriophage mechanisms (Cohen et al. 2019; Bernheim et al. 2021; Morehouse et al. 2020). Viruses are very often sensed by their nucleic acids, which bear features not shared by their host cells (Barrat et al. 2016; Hartmann 2017). Specifically, eukaryotes had to adapt to emerging RNA viruses by evolving strategies to recognize such non-self genetic material. The best characterized foreign features are 1**)** double-stranded RNA (dsRNA) structures and 2**)** triphosphate on 5′-ends, both of which are mostly absent during host cell homeostasis but are accumulated in viral infection, either directly derived from the viral genomes or formed as the replication or transcription intermediates (Weber et al. 2006; Schlee et al. 2009; Liu et al. 2015). In fungi, plants, nematodes, and arthropods, the presence of the cytoplasmatic dsRNA triggers RNA interference (RNAi) which involves dicing dsRNA into short interfering RNAs (siRNA) by the ribonuclease III Dicer, often followed by signal amplification by RNA-dependent RNA polymerases (RdRPs) and final silencing of viral RNA by Argonaute proteins (Wang et al. 2006; Felix et al. 2011; Szittya and Burgyan 2013; Lewis et al. 2018). In vertebrates, dsRNA is detected by several families of pattern-recognition receptors (PRRs) which trigger downstream expression of type I interferons (IFNs) and other proinflammatory cytokines (Hartmann 2017). Retinoic acid-inducible gene I (RIG-I)-like receptors (RLRs) are a family of metazoan-specific ATP-dependent DExD/H box RNA helicases that function as the major cytoplasmic PRRs binding dsRNA (Kang et al. 2002; Yoneyama et al. 2004, 2005) (fig. 1*a*). In vertebrates, ligands of RIG-I and its paralog melanoma differentiation-associated protein 5 (MDA5) include short, blunt-end dsRNA with 5′-di- and triphosphate (Hornung et al. 2006; Pichlmair et al. 2006; Schlee et al. 2009; Schmidt et al. 2009; Goubau et al. 2014; Ren et al. 2019) and long irregular dsRNA (Kato et al. 2006, 2008; Pichlmair et al. 2009; Peisley et al. 2012), respectively. Caspase activation and recruitment domains (CARDs) of RIG-I and MDA5 (fig. 1*b*) are necessary for regulation, oligomerization, and subsequent interaction with adaptor molecules to trigger downstream effector cascades (Rehwinkel and Gack 2020). Absence of the CARD domain in the third vertebrate RLR—Laboratory of Genetics and Physiology 2 (LGP2)—prevents signal transduction and is correlated with its dual regulatory functions (Rodriguez et al. 2014).

Although RLRs have been found in many animal phyla (fig. 1*a*) and display structural conservation (fig. 1*b*), their function in invertebrate immune response remains understudied in animals other than vertebrates and nematodes, leaving a major gap in the understanding of RLRs evolution. In this study we aimed to characterize the antiviral immune response in *Nematostella vectensis*, a model organism of phylum Cnidaria (sea anemones, corals, jellyfish, and hydroids) separated from its sister group Bilateria (the majority of extant animals, including vertebrates and nematodes) by >600 My of evolution (Technau and Steele 2011; Layden et al. 2016). Due to the current absence of any cell lines or well-characterized viruses capable of naturally infecting *N. vectensis*, we tested its response to commonly used viral dsRNA mimics. We observe in this cnidarian a strong immune response triggered by long, but not short 5′-triphosphate-bearing dsRNA which supports our phylogenetic analyses of RLRs. We show that both *N. vectensis* RLRs are likely to take part in the antiviral immune response and that one of them shows affinity to long dsRNA. Finally, knockdown (KD) of this RLR results in impaired expression of putative downstream effectors suggesting its key role in initiating immune response to dsRNA.

## Results

### Ancestral RLRs Duplication Likely Predates the Bilateria–Cnidaria Split

In order to gain a better understanding of the evolutionary fate of RLRs and the position of *N. vectensis* homologs within the family of these viral nucleic acid sensors, we reconstructed previous phylogenetic trees with an addition of numerous recently available sequences. Instead of including other distantly related DExD/H helicases, such as dsRNA-specific endoribonuclease Dicer or elongation initiation factor 4A (eIF4A), we performed phylogenetic analysis exclusively of RLRs with the sequences of sponges, one of the first two metazoan phyla to diverge (Whelan et al. 2017; Kenny et al. 2020), set as an outgroup (fig. 1*a*). Similar to previous studies (Zou et al. 2009; Mukherjee et al. 2014), we have not identified any RLR homologs in non-nematode ecdysozoans, Placozoa and Ctenophora. Within Cnidaria, we identified RLR sequences in Hexacorallia (sea anemones and stony corals) whereas they are absent in the Medusozoa clade, clearly indicating a loss. Interestingly, unlike in previous studies (Zou et al. 2009; Mukherjee et al. 2014), we observed a well-supported clustering of all hexacorallian RLRs with the bilaterian MDA5/LGP2 and together, they form a sister group to bilaterian RIG-I sequences. This unexpected finding suggests that, in contrast to the previous hypothesis (Sarkar et al. 2008), RLRs paralogs duplicated before the split of Bilateria and Cnidaria and all cnidarian RLR paralogs originated from a MDA5/LGP2 ancestral protein. Furthermore, both *Nematostella* CARD-containing protein sequences—NveRLRa and NveRLRb (henceforth referred to as RLRa and RLRb)—are positioned in separated clades with orthologs from other sea anemones suggesting their ancient duplication predating sea anemone divergence and most likely functional non-redundancy. Clustering of RLRb sequences of sea anemones within one of the clades of stony corals which have split 320 Ma (Quattrini et al. 2020) further supports the hypothesis of an ancient sub- or neofunctionalization of the sea anemone RLRs.

### Lack of Response in *Nematostella* to RIG-I-Specific Ligand

**Fig. 2.**
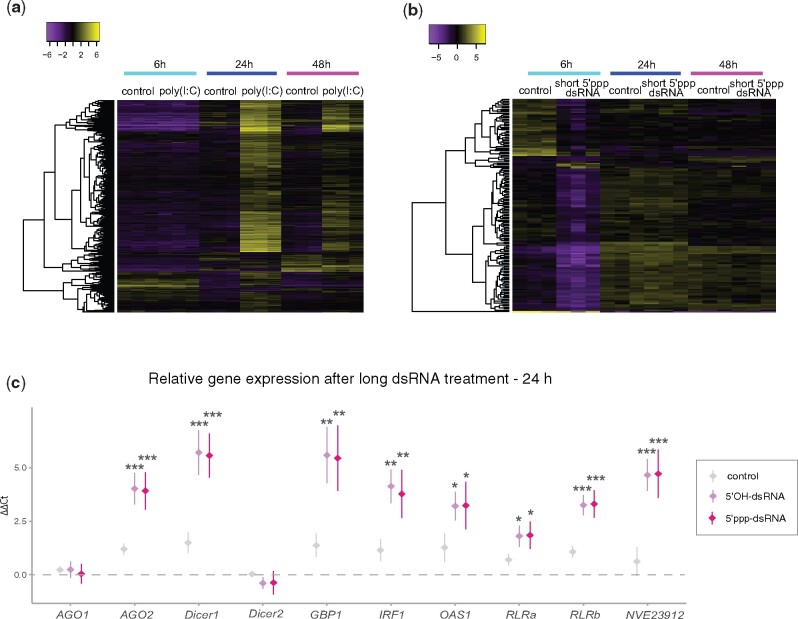
Differential gene expression after microinjections of viral mimics. Heatmap of DEGs upon administration of (*a*) poly(I:C) versus 0.9% NaCl serving as a control, and (*b*) short dsRNA with 5′-triphosphate and short dsRNA with 5′-hydroxyl group serving as a control. The heatmaps present row-centered log2 values of trimmed mean of M values (TMM). (*c*) Results of RT-qPCR measuring the expression of putative immune-related genes in response to long 5′ppp-dsRNA and long 5′OH-dsRNA. Plotted values are mean ΔΔCt relative to the uninjected animals group (dashed gray line) ± SD. All comparisons were done by one-way ANOVA with Tukey’s HSD post-hoc test. Significance level is shown for pairwise comparisons to the mock injected group.

**Fig. 3.**
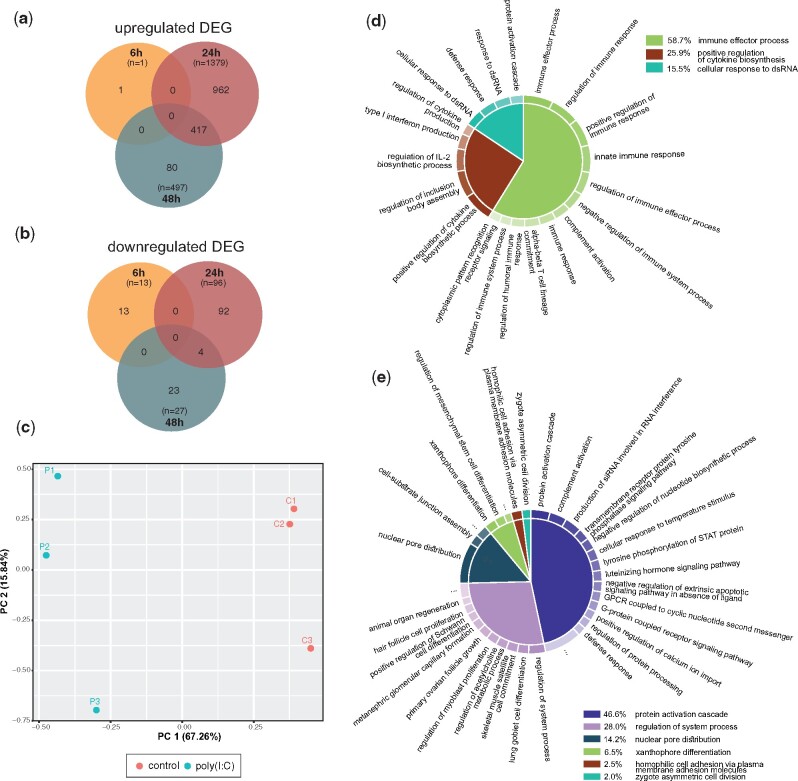
Signature of the innate immune response to poly(I:C). Venn diagram of DEGs which were (*a*) upregulated and (*b*) downregulated after poly(I:C) administration. (*c*) PCA plot representing whole transcriptome of poly(I:C)-injected animals at 24 hpi. (*d*) GO terms enrichment results of DEG upregulated at 24 hpi and (*e*) 48 hpi; semantical redundancy was previously removed with REVIGO (Supek et al. 2011). However, the list of GO terms was not manually modified to avoid any biases in the analysis results. This is reflected by the presence of some of the canonical vertebrate-specific terms. Size of the slices of GO term charts is derived from log10 *P*-value for each significantly enriched GO term and scaled to sum up to 100%.

To test whether our observation that *Nematostella* RLRs are more closely related to Bilateria MDA5 receptor is correlated with functional conservation, we decided to first employ known ligand affinity and test *Nematostella* response to MDA5 and RIG-I-specific ligands. To this end, we microinjected *N. vectensis* embryos with polyinosinic:polycytidylic acid *(*poly(I:C)), a mimic of long viral dsRNA and a potent agonist of MDA5 (Kato et al. 2006, 2008), and a short dsRNA 19-mer with 5′-triphosphate group (short 5′ppp-dsRNA) which is known to be detected by RIG-I (Schlee et al. 2009; Schmidt et al. 2009). Analysis of differentially expressed genes (DEG) upon the treatments with viral mimics revealed a strong response to poly(I:C) (fig. 2*a* and supplementary file S1 and fig. S1*d* and *e*, Supplementary Material online) with a peak of the differential expression at 24 h post-injection (hpi) accounting for 67.26% of variance revealed by principal component 1 (fig. 3*c*). Among three different time points, we have observed an almost complete lack of transcriptomic response at 6 hpi (*n* of DEG = 14) which agrees with a low transcript abundance at the onset of zygotic transcription in *Nematostella* (Helm et al. 2013). Both at 24 and 48 hpi (*n* of DEG = 1,475 and 524, respectively) the majority of DEG were upregulated (fig. 3*a* and *b*) which is a common pattern of the innate immune response to viral ligands (Andresen et al. 2020). As one replicate from poly(I:C)-treated libraries differed mildly from the other replicates, we repeated the differential expression analysis while excluding the possible outlier. Importantly, no significant difference was found between the analyses of duplicates and triplicates (supplementary fig. S2*a*–*c*, Supplementary Material online). In contrast to the poly(I:C) treatment, the transcriptomic response to vertebrate RIG-I-specific dsRNA ligand revealed a striking lack of signature of the antiviral immune processes (only 154, 0, and 1 DEGs at 6, 24 and 48 h, respectively; fig. 2*b* and supplementary file S1 and fig. S1*a*–*c*, Supplementary Material online) despite being applied at 90- to 180-fold higher concentration compared with concentrations used for vertebrates, suggesting that unlike in vertebrates (Wang et al. 2013; Kulkarni et al. 2014; Chen et al. 2016), a triphosphate group on 5′-blunt-end of short dsRNA is not triggering an immune reaction in *N. vectensis*. To evaluate whether the insensitivity to 5′-triphosphate group could be restricted to very short dsRNA molecules, we performed additional relative expression analysis of putative immune-related genes after microinjections of long dsRNA (720 bp) with and without 5′-triphosphate. Similarly to poly(I:C), both variants of long dsRNA induced a strong response at 24 hpi (fig. 2*c*) which decreased significantly at 48 hpi (supplementary file S4, Supplementary Material online). Importantly, we have not observed any statistically significant difference between the response to long dsRNA with 5′-triphosphate or hydroxyl groups (supplementary file S4, Supplementary Material online), indicating that it is length rather than the presence of the triphosphate which is a key factor for initiating antiviral response in *Nematostella*.

Results of the gene-set enrichment analysis (GSEA) revealed the abundance of gene ontology (GO) terms related to the innate immunity and strengthened our inference on strong antiviral response triggered by poly(I:C) at 24 hpi (fig. 3*d* and supplementary file S2, Supplementary Material online) and to a lesser extent at 48 hpi (fig. 3*e* and supplementary file S2, Supplementary Material online). However, it should be noted that multiple GO terms are associated with single gene, which might lead to overrepresentation of specific GO term groups. Importantly, the vast majority of responding genes at the later stage overlaps with the upregulated genes of the former one (fig. 3*a*) and shows a clear decrease in the expression level (supplementary fig. S2*d* and *e*, Supplementary Material online), suggesting a continuous attenuating immune response. In all tested groups enriched GO terms contained many vertebrate-specific terms, which were not removed to avoid introducing a bias. Thus, we had to treat them as an approximation to a true gene function. Although the GSEA for the short 5′ppp-dsRNA had not revealed enriched GO terms which would pass the statistical threshold likely due to the low DEG abundance (supplementary file S2, Supplementary Material online), we decided to examine the only DEG group responding to this treatment, that is, genes downregulated at 6 hpi (fig. 2*b*). Identified GO term groups were predominantly related to the early-stage development (supplementary fig. S1*f*, Supplementary Material online) which led us to the hypothesis that the presence of very high molarity of charged compounds might either directly or indirectly interfere with the onset of zygotic transcription, possibly by altering the cellular pH or disrupting physiological processes through the divalent cations chelating activity (Draper 2004).

### Response to Poly(I:C) Reveals Patterns of Both Invertebrate and Vertebrate Antiviral Innate Immunity

Among poly(I:C)-upregulated genes at 24 hpi we identified both of *Nematostella RLR*s, with more significant increase for *RLRb (*edgeR log_2_ fold change (FC) = 3.275, false discovery rate (FDR) = 1.54e-20) than for *RLRa* (score below the fold change threshold, i.e., edgeR log_2_FC = 1.864, FDR = 6.55e-09). This increase suggests a possibility of a positive feedback loop similar to that observed in vertebrate antiviral immune response where dsRNA sensors are among the genes which expression is further induced by the downstream signaling (Schneider et al. 2014). Moreover, many genes linked to RNAi (e.g., *Dicer1*, *AGO2*, *RdRP1-3*) and numerous homologs of genes involved in antiviral innate immune response in both vertebrates and invertebrates animals (Hartmann 2017; Wang and He 2019) *(*e.g., Interferon regulatory factors (*IRFs*), RNase L, guanylate-binding proteins (*GBP*s), 2′-5′-oligoadenylate synthetase 1 (*OAS1*), nuclear factor kappa-light-chain-enhancer of activated B cells (*NF-κB*), radical SAM domain-containing 2 (*viperin*), to mention a few, supplementary file S1, Supplementary Material online) were also detected. Interestingly, both Dicer and AGO have two paralogs in the *N. vectensis* genome (Moran et al. 2013) and the remaining genes, that is, *Dicer2* and *AGO1* were not differentially expressed in response to poly(I:C) and long dsRNA (fig. 2*c* and supplementary file S1, Supplementary Material online). Although Dicer2 function remains unknown, we have previously shown that AGO1 is mainly involved in gene regulation as its cargo is restricted to miRNA, whereas AGO2 carries both miRNA and siRNA (Fridrich et al. 2020). Interestingly, we observed a significant upregulation from a previously undescribed factor (gene symbol: NVE23912) which is a cysteine-rich sequence (11 cysteine residues) with a predicted signal peptide and no significant homology to any known genes. Our search for homologs in Transcriptome Shotgun Assembly (TSA) and NCBI nr databases revealed that it is likely a secreted hexacorallian-specific protein (supplementary fig. S3, Supplementary Material online) which resembles pattern of proteins under strong selective pressure displayed by the high conservation limited to the cysteine positions. Altogether, the described features make it a good candidate for further functional studies which could validate whether this novel factor is playing an important role in the innate immunity of *N. vectensis* and possibly other members of Hexacorallia.

To get wider view of the nature of poly(I:C)-induced DEG, we examined promoter sequences of the induced genes by two different approaches. First, we screened the coding strand for the presence of the TATA-box in both the close proximity to the transcription starting site (TSS) (38 bp) and a more permissive screening window (100 bp upstream and 100 bp downstream of TSS). It has been previously suggested that mammalian immune-related genes that are rapidly diverging and exhibit greater levels of expression variability across individual cells, such as cytokines and chemokines, share a common promoter architecture enriched in TATA-boxes (Hagai et al. 2018). Interestingly, *N. vectensis* displays a significant increase in abundance of TATA-box elements in poly(I:C)-upregulated genes when searching both window sizes which seems to correlate with the level of genes inducibility (supplementary fig. S4*a* and *b* and supplementary file S3, Supplementary Material online). Within protein sequences of TATA-box containing genes, we predicted a similar enrichment of signal peptides suggesting that many of these proteins might be involved in secretory pathways (supplementary fig. S4*c* and *d* and supplementary file S3, Supplementary Material online). Furthermore, the search of known transcription factor binding sites (TFBS) revealed numerous motifs known to be involved in regulating transcription of antiviral immune-related genes in vertebrates such as those recognized by STATs, IRFs, NF-_**κ**_B, or members of ETS family (Gallant and Gilkeson 2006; Zaslavsky et al. 2010; Chiang and Liu 2018; Seifert et al. 2019) (supplementary file S3, Supplementary Material online). In order to circumvent the limitation of using the vertebrate motif matrix, we scanned the *N. vectensis* genome for the presence of the homologs of vertebrate immune-related transcription factors. Importantly, we have identified numerous candidate homologs of these factors in the *N. vectensis* genome among which a large group showed upregulation in response to poly(I:C) treatment supporting the notion that they might play role in orchestrating the observed immune response (supplementary file S3, Supplementary Material online).

### Role of RLRs in Detecting Long dsRNA

**Fig. 4.**
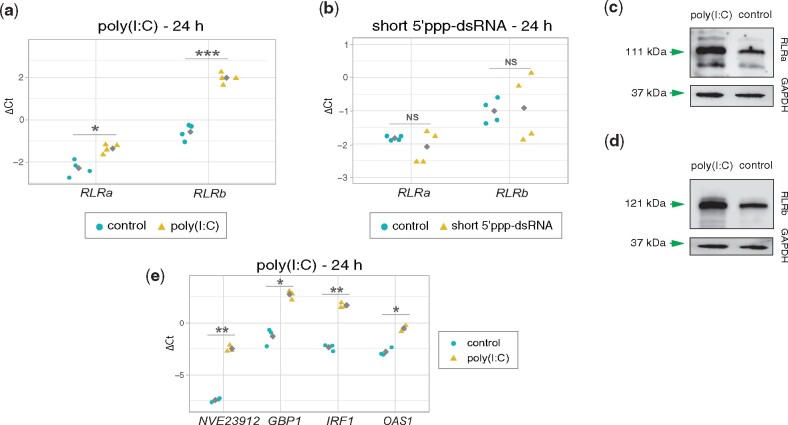
Response of *Nematostella* putative dsRNA helicases and immune-related genes to the mimics of viral ligands. *RLR*s mRNA expression level measured by RT-qPCR in response to (*a*) poly(I:C) and (*b*) short 5′ppp-dsRNA. Gray squares represent mean values. Western blot validation of (*c*) RLRa and (*d*) RLRb protein level in response to poly(I:C) at 48 hpi. (*e*) The expression level of putative immune-related genes identified as DEG after poly(I:C) treatment. Significance level for (*a*), (*b*), and (*e*) was assessed by paired two-tailed Student’s *t*-test; *P-value < 0.05, **P-value < 0.01, ***P-value < 0.001; NS, not significant.

To confirm the results of our RNA-seq DEG analysis, we assayed gene expression in independent biological replicates. RT-qPCR analysis at 24 hpi validated the upregulation of *RLR*s (relative expression_*RLRa*_ = 1.98, 95% CI, 1.042–3.494, *P*-value = 0.0425, relative expression_*RLRb*_ = 5.795, 95% CI, 3.992–8.411, *P*-value = 0.000643) (fig. 4*a*), as well as several other putative immune-related genes (fig. 4*e* and supplementary file S4, Supplementary Material online) in response to poly(I:C) treatment, and an unaffected expression level of *RLR*s transcripts when treated with short 5′ppp-dsRNA (relative expression_*RLRa*_ = 0.815, 95% CI, 0.468 –1.420, *P*-value = 0.325, relative expression_*RLRb*_ = 1.071, 95% CI, 0.528–2.173, *P*-value = 0.778) (fig. 4*b*). Importantly, we also tested the *RLR*s mRNA levels in response to the control treatments and confirmed lack of significant background upregulation which could bias our inference (supplementary fig. S5*a* and *b*, Supplementary Material online). To confirm these results at the protein level, we generated custom polyclonal antibodies against *N. vectensis* RLRs for which specificity has been characterized beforehand. RLR levels were tested at 48 hpi in order to diminish the effect of maternally deposited proteins. The result of Western blot confirmed strong upregulation of both RLRs after poly(I:C) stimulation (fig. 4*c* and *d*), which correlates with the increased transcript abundance.

**Fig. 5.**
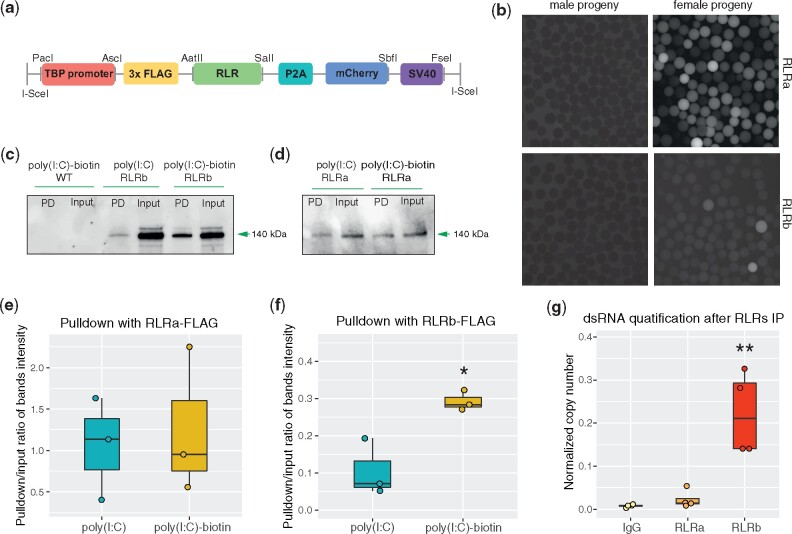
RLRs affinity to dsRNA. (*a*) Schematic representation of the FLAG-*RLR* construct (7,071 and 7,643 bp for *RLRa* and *RLRb*, respectively) used for transgenesis. TBP promoter, self-cleaving P2A sequence, mCherry gene, and polyadenylation signal SV40 are also shown. (*b*) Maternal deposition of the FLAG-RLR observed after crossing transgenic females (right panels) with WT males; fluorescent protein is missing in transgenic male progeny (left panels). (*c*) Results of poly(I:C)-biotin in vitro binding assay showing affinity of FLAG-RLRb but not (*d*) FLAG-RLRa to poly(I:C) detected with the monoclonal mouse anti-FLAG M2 antibody (Sigma-Aldrich) in Western blot; PD, pulldown. Assay was repeated in three biological replicates (supplementary file S5, Supplementary Material online) and a representative result for each RLR is shown in the figure. (*e*) Quantification of bands intensity obtained in the pulldown experiment of RLRa and (*f*) RLRb. (*g*) Results of copy number quantification of mCherry dsRNA after RLRs immunoprecipitation with custom polyclonal antibodies specific to each of the native proteins. Significance level is shown for pairwise comparisons to the control group: *P-value < 0.05, **P-value < 0.01, ***P-value < 0.001; NS, not significant.

Next, we aimed to examine the ability of RLRs to bind poly(I:C). To this end, we generated two *N. vectensis* transgenic lines, each expressing FLAG-tagged *RLR* and a fluorescent mCherry gene under a ubiquitous promoter of the TATA-Box Binding Protein (TBP) gene (fig. 5*a*). Progeny of F_1_ female heterozygotes and wild-type animals was collected directly after fertilization (0 h) and the presence of maternally deposited FLAG-tagged RLRs was confirmed (fig. 5*b*). In vitro binding assays of poly(I:C) covalently linked to biotin on wild-type protein extracts confirmed specificity of mouse FLAG antibody (fig. 5*c*). The results of the in vitro poly(I:C) binding on the transgenic lines revealed a significant enrichment of RLRb in poly(I:C)-biotin pulldown samples *(*mean_poly(I:C)_ = 0.1052, mean_poly(I:C)-biotin_ = 0.2923, *P*-value = 0.0394) indicating specific binding of this viral mimic by RLRb (fig. 5*c* and *f* and supplementary file S5, Supplementary Material online). Unexpectedly, no statistically significant poly(I:C) affinity was detected when assaying RLRa *(*mean_poly(I:C)_= 1.0567, mean_poly(I:__C)-biotin_ = 1.2539, *P*-value = 0.7695; fig. 5*d* and *e* and supplementary file S5, Supplementary Material online). In order to monitor how accurately the conditions of transgenic expression mimic the native proteome composition, we examined levels of RLRs in recently published mass spectrometry data spanning different developmental stages of *N. vectensis* (Columbus-Shenkar et al. 2018). Interestingly, we noticed that although RLRb displays relatively stable expression throughout the lifecycle, levels of RLRa in the unfertilized egg are below the detection threshold *(*in agreement with previous proteomic studies of *Nematostella* eggs (Lotan et al. 2014; Levitan et al. 2015)) and show significantly lower expression than RLRb across all developmental stages (supplementary fig. S6, Supplementary Material online). To test whether performing assays at an early developmental stage or RLR overexpression under the TBP promoter could distort the results of the ligand affinity, we performed an immunoprecipitation (IP) assay for both RLRs from the adult tissue extracts incubated with long 5′ppp-dsRNA and quantified the absolute copy number, using rabbit IgG antibodies as a control group (fig. 5*g*). These results confirmed that although RLRb shows affinity to long dsRNA, no enrichment in dsRNA was detected for RLRa *(F*(2,9) = 17.78, *P*-value = 0.00075, *P*-value_RLRb vs__.__IgG_ = 0.00122, *P*-value_RLRa vs__.__IgG_ = 0.92777; supplementary file S5, Supplementary Material online), which further supports the hypothesis that RLRa might carry other, possibly regulatory functions, or binds yet uncharacterized ligands.

### KD of *NveRLRb* Interferes with the In Vivo Response to Long dsRNA

**Fig. 6.**
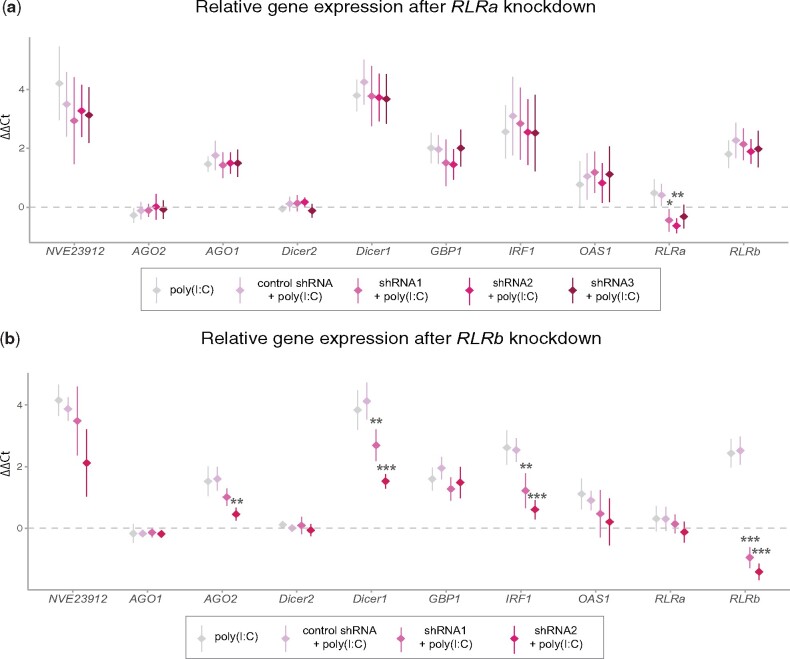
Expression of putative antiviral innate immunity-related genes in response to *RLR*s KD combined with poly(I:C) treatment. RT-qPCR results of shRNA targeting (*a*) *RLRa* and (*b*) *RLRb* genes co-injected with poly(I:C). Plotted values are mean ΔΔCt relative to the uninjected animals group (dashed gray line) ± SD. All biological replicates contained a poly(I:C)-only treated group for comparison to control-shRNA + poly(I:C) in order to validate the lack of immune response to shRNAs without the dsRNA ligand. All comparisons were done by one-way ANOVA with Tukey’s HSD post-hoc test. Significance level is shown for pairwise comparisons to the control-shRNA group: *P-value < 0.05, **P-value < 0.01, ***P-value < 0.001; NS, not significant.

Poly(I:C)-induced upregulation of *RLR*s both at the gene and the protein levels and RLRb affinity to dsRNA led us to the inference that both proteins might carry an important function in detecting viral dsRNA and hence, orchestrating downstream antiviral immune processes in *Nematostella.* To further corroborate this theory, we generated KD animals by microinjection of short hairpin RNA (shRNA) targeting three different regions of each of the *RLR*. The initial validation assays of KD efficiency and shRNAs immunogenicity revealed a strong (∼85–90%) and moderate (∼60%) effect of all *RLRb* and *RLRa* shRNAs, respectively (supplementary fig. S5*c* and *d*, Supplementary Material online), and very low impact on the expression levels of putative immune-related genes of all shRNAs (supplementary fig. S5*e*–*j*, Supplementary Material online). Due to the mild KD effect by all candidate *RLRa* shRNAs, we decided to include all three tested variants for this gene and two shRNAs for *RLRb*. Following the assumption that RLRs might act as sensors in antiviral immune response, we co-injected each shRNAs with poly(I:C) and tested at 48 hpi the mRNA levels of candidate genes previously proved to respond to the poly(I:C) treatment. The first unexpected observation was that although *RLRb* KD efficiency remained comparable to the initial screening assays (∼90%), *RLRa* KD level decreased to approximately 45% (fig. 6*a* and *b* and supplementary file S4, Supplementary Material online). Of note, none of the *RLR*s KD experiments exerted a strong and ubiquitous reciprocal effect on the other sensor. Importantly, KD of *RLRb* resulted in noticeable downregulation of both tested components of RNAi, that is, *Dicer1* and *AGO2* (significance level reached by one shRNA), as well as *IRF1*, and an apparent but not significant decrease in expression of hexacorallian-specific factor NVE23912 (fig. *6b* and supplementary file S4, Supplementary Material online). Interestingly, neither *OAS1* nor *GBP1* mRNA levels were significantly affected by the *RLRb* shRNA-poly(I:C) co-injection (fig. 6*b* and supplementary file S4, Supplementary Material online). In contrast to *RLRb* KD, response to *RLRa* shRNAs did not reveal any clear signature of the impaired downstream process in all tested genes and displayed a general pattern of high expression variation (fig. 6*a* and supplementary file S4, Supplementary Material online). Altogether, our results indicate a strong link between the presence of RLRb and the ability to initiate downstream processes involving at least two key RNAi components, that is, Dicer1 and AGO2 and a homolog of a known vertebrate IRF. Lack of effect of *RLRa* KD despite testing three shRNAs targeting different transcript regions together with the negative result of dsRNA-binding assays suggests that RLRa might carry different functions.

## Discussion

In this study, we examined transcriptomic response to three different viral dsRNA mimics in *N. vectensis* and aimed to elucidate the role of RLRs in the antiviral immune pathways. We observed lack of sensitivity to 5′-triphosphate on short and long dsRNA which supports our hypothesis about the evolution of cnidarian RLRs from an ancestral MDA5/LGP2 precursor protein (fig. 1*a*). In vertebrates, RIG-I binds to 5′-ends of dsRNA and recognizes the presence of di- and triphosphate on 2’-*O*-unmethylated nucleotide, with a strong preference to the base-paired blunt ends (Hornung et al. 2006; Pichlmair et al. 2006; Schlee et al. 2009; Schmidt et al. 2009; Goubau et al. 2014; Schuberth-Wagner et al. 2015; Ren et al. 2019). In contrast, MDA5 is known to require a stable oligomerization along the dsRNA molecule for effective downstream signaling and hence it displays a strong affinity to long molecules with at least partial stretches of dsRNA (Kato et al. 2006, 2008; Pichlmair et al. 2009; Peisley et al. 2012). Of note, poly(I:C) is known to carry 5′-diphosphate in at least a fraction of the molecules due to the synthesis process; however, uneven length of annealed strands results in single-stranded ends and long, irregular dsRNA structures (Grunberg-Manago et al. 1956). Moreover, poly(I:C) induces a similar response to long 5′OH-dsRNA, which altogether suggests that it is likely that the activation of *Nematostella* RLRs depends on the molecule length rather than the 5′-end recognition. The distinctive features of an effective RIG-I agonist have so far been mainly functionally characterized in vertebrates despite RLR homologs being found in many invertebrate genomes. Of note, the studies in the model nematode *Caenorhabditis elegans* suggested that 5′-triphosphate is not recognized by Dicer-related helicase 3 (DRH-3), an RLR homolog which plays a role in secondary siRNA production (Matranga and Pyle 2010). Interestingly, DRH-3 gains affinity to 5′-triphosphate when its nematode-specific N-terminal domain (NTD) is truncated which indicates a complex regulatory architecture and dynamic evolution of the ligand sensitivity of this helicase family (Fitzgerald et al. 2014). Therefore, further research on such nonvertebrate homologs is needed to get key insights into the evolution of dsRNA 5′-end recognition.

The fact that application of gene manipulation tools in *N. vectensis* is restricted to zygotes as well as the lack of culturable viruses infecting this species currently impede a thorough analysis of antiviral response spanning its late life stages. Yet, the transcriptomic response to poly(I:C) in early life stages revealed that many canonical vertebrate antiviral factors triggered by IFN, known as interferon-stimulated genes (Schneider et al. 2014), are also taking part in *Nematostella* immune response. Further, we observed several intriguing features of promoter region architecture such as enrichment in the TATA box sequence in poly(I:C)-upregulated genes. These elements were previously shown to display analogies in orchestrating expression of rapidly diverging and transcriptionally variable genes in phylogenetically distant groups, such as mammals (Hagai et al. 2018) and yeast (Newman et al. 2006; Tirosh and Barkai 2008). On the other hand, response to poly(I:C) and KD experiments revealed similarities to antiviral invertebrate systems and suggested a link between RLRb and the RNAi pathway. Of note, a similar level of complexity and involvement of diverse antiviral mechanisms was previously suggested for the Pacific oyster *Crassostrea gigas* (He et al. 2015; Huang et al. 2017; Lu et al. 2018; Lv et al. 2019), although the response to the canonical RLRs ligands presented here has not yet been comprehensively characterized in this molluscan species. The interdependence of RLRs and RNAi has been functionally demonstrated in *C. elegans*, where RLRs were shown to physically interact with Dicer and provide crucial assistance for RNAi machinery to produce primary and secondary antiviral siRNAs (Lu et al. 2009; Ashe et al. 2013; Guo et al. 2013). However, unlike most other bilaterian and cnidarian RLRs, the nematode receptors lack any CARD domains (fig. 1*b*) that typify action via oligomerization and signaling via downstream protein aggregation rather than association with Dicer and action via siRNAs. Importantly, there is growing evidence that virus–host interactions involve other classes of small RNAs including Dicer- and AGO-dependent microRNAs (miRNAs) (Bernier and Sagan 2018) and several studies in chordates suggested differential expression of host miRNAs in response to poly(I:C) (Wang et al. 2016; Zhang et al. 2017; Singaravelu et al. 2019; Wu et al. 2019). We have recently demonstrated that the cargo of AGO1 is restricted to miRNAs, whereas AGO2 can carry both miRNAs and siRNAs (Fridrich et al. 2020). This hints that poly(I:C)-upregulated AGO2 could function as the antiviral RNAi effector protein by slicing viral RNA through virus-derived siRNAs (viRNAs) guidance. However, recent study on viRNAs abundance in non-model invertebrates showed that this class of non-coding RNAs is hardly detectable in some species, including the sea anemone *Actinia equina* (Waldron et al. 2018). Therefore, further studies are crucial to decipher the role of RNAi components in the antiviral immune response of *N. vectensis* and other cnidarians.

The results of *RLRb* KD indicate that there are likely alternative immune cascades triggered by poly(I:C) administration which might be initiated by other dsRNA sensors. Among these, Toll-like receptors (TLRs) are obvious candidates due to their well-known role as PRRs (Kawasaki and Kawai 2014). However, the only TLR of *N. vectensis* has been recently shown to mediate immune response in NF_**κ**_B-dependent way in response to *Vibrio coralliilyticus* and flagellin (Brennan et al. 2017) which indicates its involvement in recognizing bacterial rather than viral pathogens. An intriguing question for future studies is whether RLRa is acting as a nucleic acid sensor. On one hand, the stable coexistence of two separately clustering RLRs paralogs in sea anemones (fig. 1*a*) and the clear increase in *RLRa* expression upon poly(I:C) challenge (fig. 4*a* and *c*) suggest that it is likely a functional component of antiviral immune response which might display affinity to yet uncharacterized ligands. Nonetheless, the short truncation of its helicase domain and aberrant KD patterns suggest an alternative but not mutually exclusive hypothesis that RLRa might carry some regulatory functions involved in complex feedback mechanisms.

To the best of our knowledge, our study provides the first functional insights into the role of RLRs in a non-bilaterian animal. The initial results suggest that RLRs capacity to sense 5′-end of dsRNA evolved in Bilateria, although further studies involving invertebrate RLRs will provide key answers on this matter. We show that *N. vectensis* response to a viral dsRNA mimic is characterized by high complexity and includes both vertebrate-like features, as well as invertebrate-like involvement of RNAi machinery in an RLR-dependent manner. This shows that key elements of both extant antiviral systems were already present in a cnidarian–bilaterian common ancestor. Our results lay the foundation for further functional studies on downstream effector mechanisms in *N. vectensis* which might provide crucial insights into the evolution of the antiviral immune response in Metazoa.

## Materials and Methods

### Sea Anemone Culture

*Nematostella* embryos, larvae, and juveniles were grown in the dark at 22 °C in 16‰ artificial seawater, whereas polyps were grown at 18 °C and fed with *Artemia salina* nauplii three times a week. The induction of gamete spawning was performed as previously described (Genikhovich and Technau 2009). The gelatinous egg sack was removed using 3% l-cysteine (Merck Millipore, Burlington, MA) and followed by microinjection of viral mimics or shRNAs. All *N. vectensis* individuals used in this study belonged to the common lab strain originating from Rhode River MD (Hand and Uhlinger 1992).

### Injection of Viral Mimics

To stimulate the antiviral immune response in *Nematostella*, we used three types of synthetic dsRNA. To mimic the presence of long dsRNA, we used 3.125 ng/µl of high molecular weight (HMW) poly(I:C) in 0.9% NaCl (Invivogen, San Diego, CA) with an average size of 1.5–8 kb, and 0.9% NaCl as a control. This concentration was chosen after initial titration assays which revealed that the higher concentrations (i.e., _**≥**_6.25 ng/µl) resulted in massive mortality after 48 h and aberrant zygotes morphology. The second type of ligand was a synthetic dsRNA 19-mer with 5′-triphosphate (short 5′ppp-dsRNA) and a control dsRNA 19-mer with 5′-hydroxyl group (short 5′OH-dsRNA or control), both suspended in sterile RNase-free endotoxin-free water to a final concentration of 90 ng/µl (Invivogen). The initial titration assays did not indicate any survival or morphological response to the short dsRNA ligands, so the highest possible dose of these viral mimics was used for further assays. Third type of viral ligand mimic was 0.875 ng/µl long dsRNA corresponding to eGFP sequence (720 bp long) with the 5′-end carrying triphosphate (long 5′ppp-dsRNA) or hydroxyl group (long 5′OH-dsRNA) (RiboPro, Oss, Netherlands). The concentration of long dsRNA used grossly corresponded to the molar concentration of the poly(I:C) assays. Each experiment was performed in triplicates and each biological replicate was composed of 100–150 injected zygotes per time point. Within each biological replicate zygotes were collected at 6, 24, and 48 hpi (24 and 48 hpi only for long dsRNA experiments), flash frozen in liquid nitrogen, and stored at −80 °C until processed.

### Transcriptome Library Preparation and Sequencing

Total RNA was extracted with Tri-Reagent (Sigma-Aldrich, St. Louis, MO) according to manufacturer’s protocol, treated with 2 µl of Turbo DNase (Thermo Fisher Scientific, Waltham, MA), and re-extracted with Tri-Reagent and 20 µg of RNA-grade glycogen (Thermo Fisher Scientific). The quality of total RNA was assessed on Bioanalyzer Nanochip (Agilent, Santa Clara, CA), and only samples with RNA integrity number >7 were retained. Libraries were constructed from 226 and 300 ng of total RNA from poly(I:C) and 5′ppp-dsRNA-injected samples, respectively. RNA-seq libraries were generated using SENSE Total RNA-seq Library Prep Kit v2 (Lexogen, Vienna, Austria) following the manufacturer’s protocol and sequenced on NextSeq 500 (Illumina, San Diego, CA) using single-end 75 bp chemistry.

### Raw Reads Processing and Differential Gene Expression Analysis

Quality of raw reads was assessed and visualized with FastQC software (Andrews 2010). Reads were trimmed and quality filtered by Trimmomatic with the following parameters (HEADCROP:9 LEADING:3 TRAILING:3 SLIDINGWINDOW:4:20 MINLEN:36) (Bolger et al. 2014), and the quality of the filtered reads was re-assessed in FastQC. Reads were mapped to the *N. vectensis* genome (NCBI accession number: GCA_000209225.1) (Putnam et al. 2007) with STAR alignment program (version 2.7.3a) (Dobin et al. 2013). Gene counts were obtained with RSEM (Li and Dewey 2011) (genes models, protein models, and annotations are available at: https://figshare.com/articles/Nematostella_vectensis_transcriptome_and_gene_models_v2_0/807696, last accessed August 10, 2019). Differential gene expression analysis was carried out with edgeR (Robinson et al. 2010) and DESeq2 (Love et al. 2014) implemented in the Trinity pipeline (Haas et al. 2013). Treatment samples within each time point were compared with the corresponding control samples. DEGs were defined by FDR < 0.05 and log_2_fold change ≥ 2. Only genes identified by both edgeR and DESeq2 methods were reported as differentially expressed. GO groups were identified by GSEA using GOseq Bioconductor package (Young et al. 2010) implemented in the in-built Trinity pipeline (Haas et al. 2013). An FDR cut-off of 0.05 was considered significant for the enriched or depleted GO terms. To reduce redundancy, GO terms were group based on semantic similarity using REVIGO (Supek et al. 2011) and visualized by CirGO v2.0 (Kuznetsova et al. 2019).

### shRNA Generation and KD Experiments

Three shRNA precursors from three different regions of each *RLR* gene as well as control shRNAs were designed and prepared as previously described (Karabulut et al. 2019) with minor modifications. In brief, 19bp gene targeting motif size was chosen for each shRNA (minimum GC% content > 35%). We have introduced 2–3 mismatches to the star strand, which corresponds to the coding strand, to create the bulges in shRNA precursors following the structure of native miRNA in *Nematostella* (Moran et al. 2017; Fridrich et al. 2020). Reverse complement sequence of shRNA precursors was synthesized as DNA ultramer oligo by Integrated DNA Technologies (Coralville, IA), mixed with T7 promoter primer in 1:1 ratio in a final concentration of 25 µM, denatured at 98 °C for 5min, and cooled to 24 °C. shRNAs were synthesized with AmpliScribe T7-Flash Transcription Kit (Epicentre, Charlotte, NC) for 15 h followed by 15-min treatment with 1 µl of DNase I. The in vitro transcribed products were purified using the Quick-RNA Miniprep Kit (Zymo Research, Irvine, CA). The quality and size of each precursor were checked on 1.5% agarose gel and its concentration was measured by spectrophotometer. The sequences of shRNAs precursors are provided in supplementary file S6, Supplementary Material online.

Initial screening of shRNA KD efficiency and toxicity revealed that microinjections of shRNAs of *RLRa* and *RLRb* proved effective and nontoxic at 48 hpi in 750–1,000 and 350–500 ng/µl concentration range, respectively. Three shRNAs for *RLRa* (750, 750, and 1,000 ng/µl) and two shRNAs for *RLRb* (500 ng/µl each) were microinjected to *Nematostella* zygotes in a 10 µl mixture containing additionally 3.125 ng/µl of HMW poly(I:C), 1 µl of 9% NaCl and RNase-free endotoxin-free water. Identically prepared 1,000 and 500 ng/µl of the control shRNA was included in each microinjection of *RLRa* and *RLRb* shRNAs, respectively. Moreover, in each microinjection experiment we included a subset of animals treated only with poly(I:C) 3.125 ng/µl to monitor the cytotoxic effect of shRNA control. Zygotes were collected at 48 hpi, flash frozen in liquid nitrogen, and stored at −80 °C until further processed.

### Reverse-Transcription Quantitative PCR

To validate the results of the RNA-seq, KD experiments, long dsRNA injection, and RLRs-IP, we assayed the expression levels of several candidate immune-related genes from the mammalian RLR pathway (*RLRa*, *RLRb*, *OAS1*, *IRF1*, *GBP1*), RNAi pathway (*Dicer1*, *Dicer2*, *AGO1*, and *AGO2*), and a representative of hexacorallian-specific gene (NVE23912) by reverse-transcription quantitative PCR (RT-qPCR) at 24 hpi (RNA-seq) or 48 hpi (KD experiments) or both time points (long dsRNA injection). Three to five biological replicates were used to validate the results of transcriptomics, poly(I:C)-shRNAs experiments and long dsRNA injections (detailed number of replicates is shown in supplementary file S4, Supplementary Material online), whereas one biological replicate was used to assess the efficiency and background immune response to shRNAs and poly(I:C) control. RNA was extracted from injected embryos following the same protocol used for RNA-seq libraries construction and the 500 ng of RNA was converted into cDNA in a 20 μl reaction. cDNA was constructed using SuperScript III (Thermo Fisher Scientific) for RNA-seq validation and RLRs-IP results and iScript cDNA Synthesis Kit (Bio-Rad, Hercules, CA) for KD experiments and long dsRNA injection, according to the manufacturer’s protocol. Real-time PCR was prepared with Fast SYBR Green Master Mix (Thermo Fisher Scientific) on the StepOnePlus Real-Time PCR System v2.2 (ABI, Thermo Fisher Scientific). The qPCR mixture contained cDNA template (1 μl), 2× Fast SYBR Green Master Mix (5 μl), 10 μM primers (1 μl), and nuclease-free water to make up 10 μl total volume. qPCR thermocycling conditions were 95 °C for 20 s, 40 cycles of 95 °C for 3 s, and 60 °C for 30 s. Melt curve analysis was initiated with 95 °C for 15 s and performed from 60 to 95 °C in 0.5 °C increments. The expression levels of tested genes were normalized to the NVE5273 gene (ΔCt = Ct_reference gene_ − Ct_gene of interest_) which its stable expression level was previously demonstrated (Columbus-Shenkar et al. 2018), and the relative expression was calculated by the 2^ΔΔCt^ method. The significance level was calculated by applying paired two-tailed Student’s *t*-test to ΔCt values for each of the pairwise comparisons or, in case of >2 groups experiments, ANOVA test followed by Tukey’s HSD post hoc procedure with a multiple test correction applied to ΔΔCt values. For RLRs-IP experiment, a standard curve of mCherry amplicon was prepared and used for the absolute copy number quantification. Sequences of all primers and the values obtained in primer calibration assays are shown in supplementary file S6, Supplementary Material online. Calibration curves for each primer pair and the example melt curves used to assess primers specificity are shown in supplementary figure S7, Supplementary Material online.

### Antibody Generation

For RLRa and RLRb Western blots following poly(I:C) stimulation, we used custom polyclonal antibodies raised against recombinant fragment antigens generated by rabbits’ immunization (GenScript, Piscataway Township, NJ). Each recombinant fragment was injected into three rabbits. After the third round of immunization, preimmune and postimmune sera were sent to us for screening by Western blot against *Nematostella* lysate to identify sera specifically positive for RLRa and RLRb (bands of ∼111 and ∼121 kDa, respectively). Finally, the antigens were used by the company for affinity purification from the relevant rabbits. Amino acid sequences of RLRa and RLRb fragments used for immunization are presented in supplementary file S6, Supplementary Material online.

### Western Blot

Equal amounts of protein were run on 4–15% Mini-PROTEAN TGX Precast Protein Gel (Bio-Rad) followed by blotting to a polyvinylidene fluoride membrane (Bio-Rad). Next, the membrane was washed with TBST buffer (20 mM Tris pH 7.6, 150 mM NaCl, 0.1% Tween 20) and blocked (5% skim milk [BD, Franklin Lakes, NJ] in TBST) for 1 h on the shaker at room temperature. Polyclonal antibody against RLRa or RLRb, monoclonal mouse anti-FLAG M2 antibody (Sigma-Aldrich), or monoclonal mouse anti-GAPDH (Abcam, Cambridge, United Kingdom) serving as loading control was diluted 1:1,000 in TBST containing 5% BSA (MP Biomedicals, Irvine, CA) and incubated with the membrane in a sealed sterile plastic bag at 4 °C overnight. The membrane was washed three times with TBST for 10 min and incubated for 1 h with 1:10,000 diluted peroxidase-conjugated antimouse or antirabbit antibody (Jackson ImmunoResearch, West Grove, PA) in 5% skim milk in TBST. Finally, the membrane was washed three times with TBST and detection was performed with the Clarity Max ECL kit for pulldown experiments (Bio-Rad) and Clarity ECL kit for all other experiments (Bio-Rad) according to the manufacturer’s instructions and visualized with a CCD camera of the Odyssey Fc imaging system (Li-COR Biosciences, Lincoln, NE). Size determination was carried out by simultaneously running Precision Plus Protein Dual Color Protein Ladder (Bio-Rad) and scanning at 700 nm wavelength.

### Cloning and Transgenesis

Synthetic genes (Gene Universal, Newark, DE) including CDS of *RLRa* and *RLRb* (scaffold_15:1090025-1101489 and scaffold_40:683898-697394, respectively), self-cleaving porcine teschovirus-1 2A sequence (P2A) (Kim et al. 2011), and mCherry sequence (Shaner et al. 2004) were amplified with Q5 Hot Start High-Fidelity DNA Polymerase (New England Biolabs, Ipswich, MA), visualized on 1% agarose gel, and purified by NucleoSpin Gel and PCR Clean-up (Macherey-Nagel, Düren, Germany). Following digestion with restriction enzymes, PCR fragments were ligated to a pER242 (Renfer and Technau 2017) vector containing a TBP promoter previously proved to drive ubiquitous expression in *Nematostella* (Admoni et al. 2020), three N-terminal FLAG tags, and SV40 polyadenylation signal. Plasmids were transformed into the *Escherichia coli* DH5α (New England Biolabs) strain and outsourced for Sanger sequencing (HyLabs, Rehovot, Israel). Each *RLR* plasmid was subsequently injected into *N. vectensis* zygotes along with the yeast meganuclease I-*Sce*I (New England Biolabs) to enable genomic integration (Renfer et al. 2010; Renfer and Technau 2017). Transgenic animals were visualized under an SMZ18 stereomicroscope equipped with a DS-Qi2 camera (Nikon, Tokyo, Japan) and positive animals were reared to the adult stage. At approximately 4 months old F_0_ individuals were induced for gametes and crossed with wild-type animals to generate F_1_ FLAG-tagged TBP::RLR::mCherry heterozygotes. Positive F_1_ individuals were selected and grown to the adult stage. For the in vitro binding assay, only F_1_ females descending from a single F_0_ founder of each RLR line were used. Sequences of all used primers are provided in supplementary file S6, Supplementary Material online.

### In Vitro Binding Assay

Maternal deposition of FLAG-tagged TBP::RLR::mCherry transgene in F_2_ animals was visualized under an SMZ18 stereomicroscope equipped with a DS-Qi2 camera (Nikon) and confirmed by Western blotting. Following fertilization with wild-type gametes, F_2_ FLAG-tagged TBP::RLR::mCherry and wild-type zygotes were treated with 3% l-cysteine (Merck Millipore), washed and snap frozen in liquid nitrogen. Next, animals were mechanically homogenized in the following lysis buffer: 50 mM Tris-HCl (pH 7.4), 150 mM KCl, 0.5% NP-40, 10% glycerol, protease inhibitor cOmplete ULTRA tablets (Roche, Basel, Switzerland) and Protease Inhibitor Cocktail Set III, EDTA-Free (Merck Millipore). Protease inhibitors were added fresh just before use. After 1 h rotation in 4 °C the samples were centrifuged at 16,000 × g, 15 min, 4 °C and supernatant was collected. Protein concentration was measured using Pierce BCA Protein Assay Kit (Thermo Fisher Scientific). Next, the lysate was precleared as following: 100 µl of streptavidin magnetic beads (New England Biolabs) was washed in 1 ml of 1× PBS for three times and the FLAG-tagged TBP::RLR/wild-type lysate was added to the washed beads. Lysis buffer was added to make up 1.2 ml and samples were incubated at 4 °C rotation for 1 h. After the incubation, the precleared lysates were collected and mixed with the HMW poly(I:C) (Invivogen) or HMW poly(I:C)-biotin (Invivogen) in the final concentration of 30 ng/ml and ATP (New England Biolabs) in the final concentration of 0.5 mM. Samples were incubated for 1 h in rotation at room temperature. Simultaneously, 100 µl of fresh streptavidin magnetic beads were blocked with wild-type lysates alike in the preclearing step. poly(I:C)-containing lysates were added to the blocked beads and incubated for 2 h in rotation at 4 °C for poly(I:C)-biotin pulldown. An amount of 100 µl was taken from each lysate before addition to the beads as input sample. After the incubation, the lysates were discarded and the beads were washed three times with 500 µl of the following wash buffer: 50 mM Tris-HCl (pH 7.4), protease inhibitor cOmplete ULTRA tablets, and Protease Inhibitor Cocktail Set III, EDTA-Free. Subsequently, 40 µl of filtered double-distilled water and 20 and 50 µl of Blue Protein Loading Dye (New England Biolabs) were added to the beads and the inputs, respectively. The samples were heated at 100 °C for 8 min and placed on ice for 1 min, then pulldown samples were centrifuged 1 min at 21,000 × g at 4 °C, and the supernatant was collected for Western blot.

### RLRs Immunoprecipitation

SureBeads Protein A Magnetic Beads (Bio-Rad) were washed five times in 1 ml of 1× PBS and 5 µg of anti-RLRa, anti-RLRb (GenScript), or total Rabbit IgG (Sigma-Aldrich) antibodies were added to the beads with 1.4 ml of 1× PBS. Samples were left on rotation at 4 °C for overnight. Adult animals (mixed males and females) were starved for 3 days and snap frozen in liquid nitrogen. Next, animals were mechanically homogenized in the following lysis buffer: 5 mM Tris-HCl (pH 7.4), 150 mM KCl, 0.5% NP-40, 1 mM DTT, protease inhibitor cOmplete ULTRA tablets and Protease Inhibitor Cocktail Set III, EDTA-Free. Protease inhibitors were added fresh just before use. After 2 h rotation in 4 °C (vortexed briefly every 30 min), the samples were centrifuged at 16,000 × g, 15 min, 4 °C and supernatant was collected. Protein concentration was measured in 1:20 diluted samples using Pierce BCA Protein Assay Kit (Thermo Fisher Scientific). Next, the lysate was precleared as following: 100 µl of SureBeads Protein A Magnetic Beads (Bio-Rad) was washed in 1 ml of 1× PBS for three times and the tissue lysate was added to the washed beads. Lysis buffer was added to make up 1.4 ml and samples were incubated at 4 °C rotation for 1 h. Next, the precleared lysates were collected and mixed with the 0.455 ng/ml (final concentration) of mCherry 5′ppp-dsRNA (RiboPro) and ATP (New England Bioloabs) in the final concentration of 0.5 mM and incubated for 1 h at room temperature with rotation. After the incubation with dsRNA, the mixtures were added to the beads-bound antibodies and incubated at 4 °C for 2 h with rotation. After the incubation, the lysates were discarded and the beads were washed five times with 500 µl of the following wash buffer: 50 mM Tris-HCl (pH 7.4), 300 mM NaCl, 5 mM MgCl_2_, 0.05% NP-40, protease inhibitor cOmplete ULTRA tablets, and Protease Inhibitor Cocktail Set III, EDTA-Free. After the last wash beads were directly subjected to RNA extraction using Trizol (Thermo Fisher Scientific) according to the manufacturer’s protocol with 20 µg of RNA-grade glycogen (Roche) added at the isopropanol precipitation stage.

### Phylogenetic Analysis

To construct an informative phylogenetic tree, we selected representatives of major groups carrying RLRs: vertebrates (a fish, an amphibian, and a mammal), two nonvertebrate chordates (a lancelet and a lamprey), nematodes (*C. elegans* and *A. suum*), four lophotrochozoans (an annelid, a brachiopod, a flatworm and a mollusk), and hexacorallians (three sea anemones, each representing a different major sea anemone clade and two-reef building corals). Sponge RLRs sequences were chosen as an outgroup. The RLRs amino acid sequences were aligned using MUSCLE (Edgar 2004) and low certainty alignment regions were removed by TrimAl (Capella-Gutierrez et al. 2009) using the –automatic1 for heuristic model selection. The maximum-likelihood phylogenetic trees were constructed using IQ-Tree (Nguyen et al. 2015) with the LG+F+R5 model which was the best-fitting model according to the Bayesian information criterion. Support values of the ML tree were calculated by three different methods: 1,000 ultrafast bootstrap replicates (Minh et al. 2013), 1,000 replicates of the Shimodaira–Hasegawa approximate likelihood ratio test (SH-aLRT), and an approximate Bayes test (Anisimova et al. 2011). Consensus domain composition was predicted by simultaneous search in Pfam (El-Gebali et al. 2019) and NCBI Conserved Domains (Lu et al. 2020) databases run with default parameters.

Homologs of NVE23912 sequences were identified through a search in TSA and NCBI nr databases and *Nematostella* gene models. Amino acid sequences were aligned using MUSCLE (Edgar 2004) and visualized by CLC Genomics Workbench. Details of RLRs and NVE23912 homolog sequences used in the analysis are available in supplementary file S7, Supplementary Material online.

### Promoter Sequence Analysis of DEG

Analysis of promoter sequences was performed as previously described (Hagai et al. 2018) with minor modifications. In brief, coordinates of the TSS were retrieved from nveGenes.vienna130208.nemVec1.bed file. We subset the upregulated DEG identified by poly(I:C) microinjection (*n* = 1,379) and the fraction of top 10% genes (*n* = 138) and top 20% genes (*n* = 276), setting the whole transcriptome as the background (*n* = 18,831). TATA box-containing genes were identified using FIMO (Grant et al. 2011) by having at least one statistically significant match (*P*-value cut-off of <0.05) to the TATA box consensus motif (MA0108.1) retrieved from JASPAR server (Fornes et al. 2020). Due to uncertainty in TSS calling, we have scanned the coding strand in two ways: 1) narrow search included 38 bp upstream of TSS and 2) wide search spanned both 100 bp upstream and 100 bp downstream of putative TSS whenever fitted in the scaffold boundaries. To estimate motifs enrichment in the same groups, we used the nonredundant JASPAR core motif matrix (pfm_vertebrates.txt) and run AME (McLeay and Bailey 2010) in one-tailed Fisher’s exact test mode. The searching region included 500 bp upstream of the putative TSS, the first exon, and the first intron of the gene. For motif identification, the cut-off of adjusted by Bonferroni correction *P*-value <0.05 was considered statistically significant. The presence of the signal peptide in each protein sequence was predicted by SignalP 4.1 Server with default settings (Petersen et al. 2011).

## Supplementary Material

Supplementary data are available at *Molecular Biology and Evolution* online.

## Supplementary Material

msab197_Supplementary_Data
