# Supplementary material for: Functional Characterization of the Cnidarian Antiviral Immune Response Reveals Ancestral Complexity
Source: Mol Biol Evol. 2021 Jun 28;38(10):4546–61. doi: 10.1093/molbev/msab197 (PMC8476169; doi:10.1093/molbev/msab197)
Supplement: msab197_Supplementary_Data [file msab197_supplementary_data.zip › Supplementary figure legends.docx]

**Supplementary Figures**

**Functional characterization of the cnidarian antiviral immune response reveals ancestral complexity**

Magda Lewandowska^1,*^, Ton Sharoni^1^, Yael Admoni^1^, Reuven Aharoni^1^, Yehu Moran^1,*^

^1^Department of Ecology, Evolution and Behavior, Alexander Silberman Institute of Life Sciences, Faculty of Science, Hebrew University of Jerusalem, Jerusalem 9190401, Israel

^*^Correspondence: magda.lewandowska@mail.huji.ac.il (M.L.); yehu.moran@mail.huji.ac.il (Y.M.)

**Content:**

**Figure S1. Differential gene expression between control and viral mimics.** PCA plots representing whole transcriptome of **(a-c)** short 5'ppp dsRNA-injected animals and **(d-e)** poly(I:C)-injected animals assayed at different time points. **(f)** GO terms enrichment after REVIGO-based semantic similarity filtering of downregulated DEG at 6 hpi after short 5'ppp dsRNA injection. Enrichment score was defined as -(log_10_ p value).

**Figure S2. Comparison of the results of differential expression analysis done in biological duplicates and triplicates. (a)** Lack of difference in the ratio of DEGs between two tested time points; Venn diagrams showing that the majority of upregulated DEGs at **(b)** 24 hpi and **(c)** 48 hpi are shared. Decreasing expression of genes identified as DEGs in both time points when analysing biological **(d)** duplicates and **(e)** triplicates. Heatmaps present row-centered log2 values of trimmed mean of M values (TMM).

**Figure S3. Alignment of NVE23912-like homologs identified in Hexacorallia species.** Ate, *Actinia tenebrosa*, Cau,*Corynactis australis*, Dpe, *Desmophyllum pertusum* (previously *Lophelia pertusa*), Eca, *Edwardsiella carnea*, Epa, *Exaiptasia pallida*, Nve, *Nematostella vectensis*, Ofa, *Orbicella faveolate,* Pst, *Pseudodiploria strigosa*, Sca, *Scolanthus callimorphus*.

**Figure S4. Frequency of genes with predicted TATA box elements and signal peptide within poly(I:C)-induced genes.** Frequency of TATA-box positive genes were identified by search within **(a)** 100 bp upstream and 100 bp downstream of TSS, and **(b)** 38 bp upstream of TSS. Signal peptide was identified in the same **(c)** wide and **(d)** narrow search windows. Significance level was assessed by two-tailed Fisher’s exact test; * p value < 0.05, ** p value <0.01, *** p value <0.001.

**Figure S5. Background immune response to shRNA and controls for viral mimics.** Relative gene expression level of selected putative immune-related genes in response to **(a)** poly(I:C) and 0.9% NaCl, **(b)** short dsRNA with and without 5' triphosphate. Knockdown efficiency of **(c)** *RLRa* shRNAs and **(d)** *RLRb* shRNAs. Expression of putative immune-related genes in response to **(e-g)** *RLRa* shRNAs, **(h-j)** *RLRb* shRNAs. Error bars represent standard deviation of technical replicates.

**Figure S6. RLRs protein level in various developmental stages.** A graph presents at a logarithmic scale the mass spectrometry-measured iBAQ (Intensity Based Absolute Quantification) values. Error bars represent standard deviation. Data acquired from Columbus-Shenkar *et al.*^50^

**Figure S7. Assessment of RT-qPCR primer specificity and sensitivity. (a,b)** Primer calibration curves. Detailed values of standard curve obtained at the calibration step are presented in Supplementary File S6. **(c)** Example of melt curves for each targeted gene.
