# Supplementary material for: Functional Characterization of the Cnidarian Antiviral Immune Response Reveals Ancestral Complexity
Source: Mol Biol Evol. 2021 Jun 28;38(10):4546–61. doi: 10.1093/molbev/msab197 (PMC8476169; doi:10.1093/molbev/msab197)
Supplement: msab197_Supplementary_Data [file msab197_supplementary_data.zip › Supplementary Figures.pdf]

Figure S1

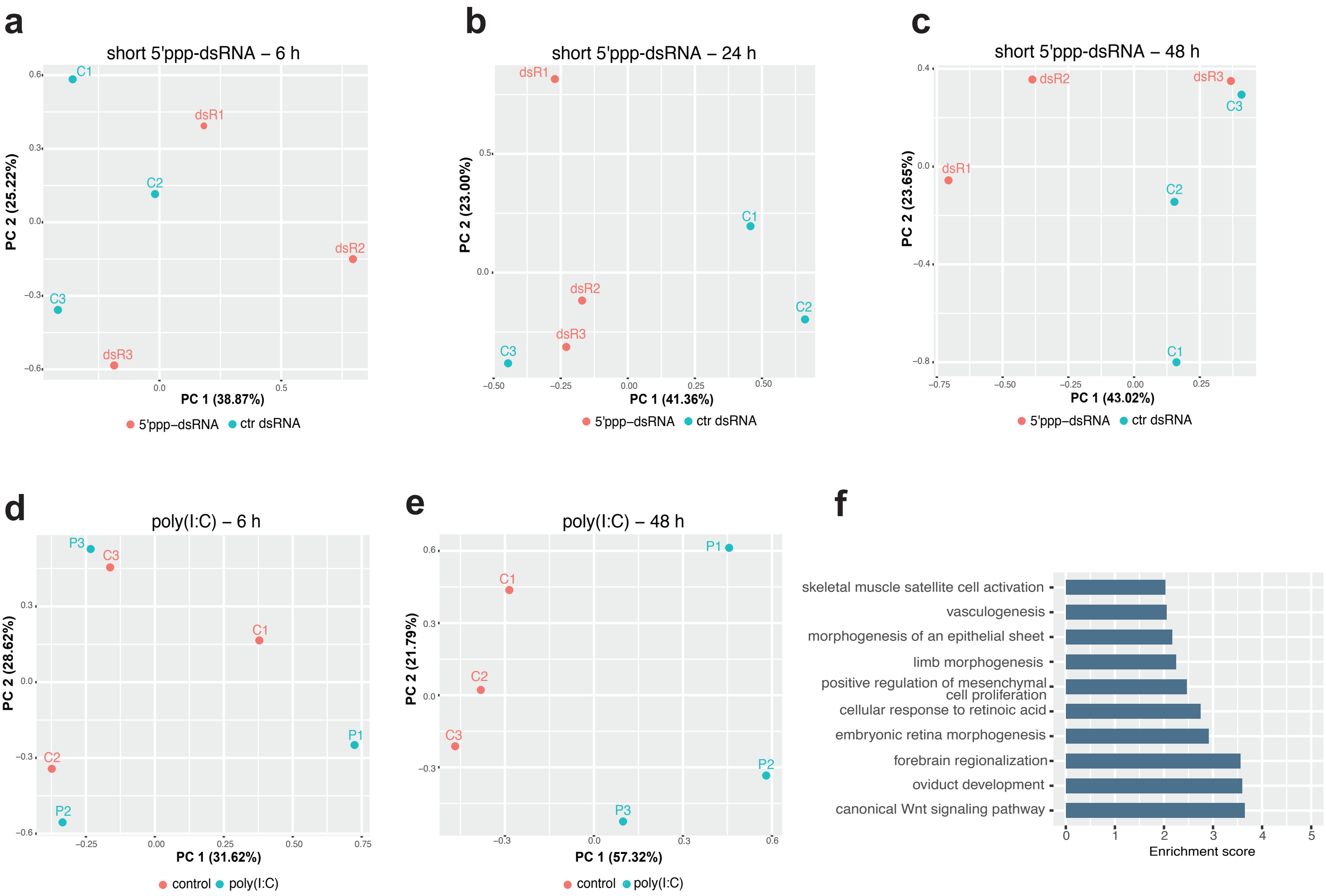

a
short 5'ppp-dsRNA – 6 h
PC 1 (38.87%)
PC 2 (25.22%)
C1
dsR1
C2
dsR2
C3
dsR3
5'ppp–dsRNA
ctr dsRNA
b
short 5'ppp-dsRNA – 24 h
PC 1 (41.36%)
PC 2 (23.00%)
dsR1
C1
dsR2
C2
dsR3
C3
5'ppp–dsRNA
ctr dsRNA
c
short 5'ppp-dsRNA – 48 h
PC 1 (43.02%)
PC 2 (23.65%)
dsR2
dsR3
C3
dsR1
C2
C1
5'ppp–dsRNA
ctr dsRNA
d
poly(I:C) – 6 h
PC 1 (31.62%)
PC 2 (28.62%)
P3
C3
C1
P1
C2
P2
control
poly(I:C)
e
poly(I:C) – 48 h
PC 1 (57.32%)
PC 2 (21.79%)
P1
C1
C2
C3
P2
P3
control
poly(I:C)
f
skeletal muscle satellite cell activation
vasculogenesis
morphogenesis of an epithelial sheet
limb morphogenesis
positive regulation of mesenchymal cell proliferation
cellular response to retinoic acid
embryonic retina morphogenesis
forebrain regionalization
oviduct development
canonical Wnt signaling pathway
Enrichment score

Figure S2

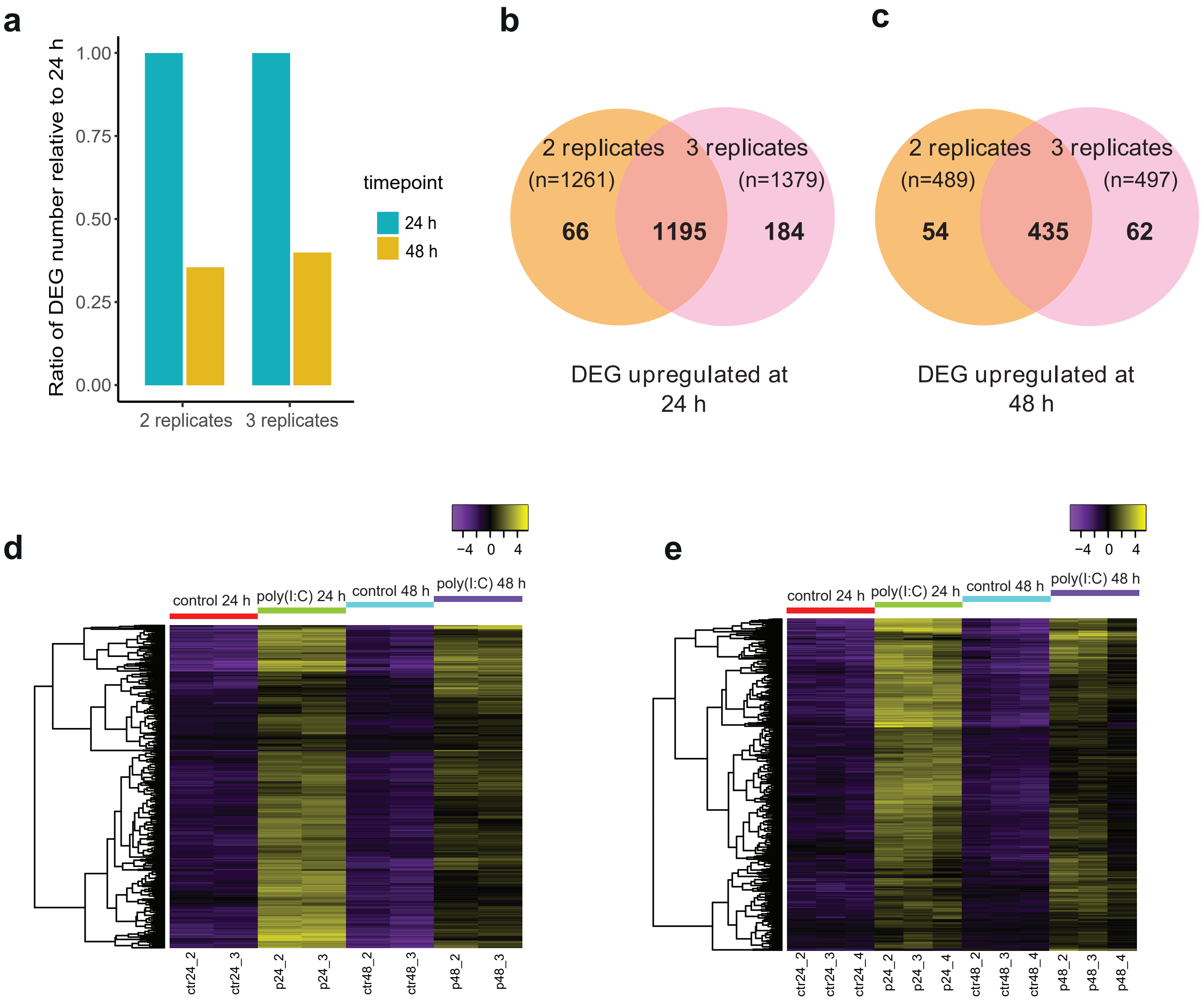
a
Ratio of DEG number relative to 24 h
1.00
0.75
0.50
0.25
0.00
2 replicates
3 replicates
timepoint
24 h
48 h
b
2 replicates
(n=1261)
3 replicates
(n=1379)
66
1195
184
DEG upregulated at
24 h
c
2 replicates
(n=489)
3 replicates
(n=497)
54
435
62
DEG upregulated at
48 h
d
−4 0 4
control 24 h
poly(I:C) 24 h
control 48 h
poly(I:C) 48 h
ctr24_2
ctr24_3
p24_2
p24_3
ctr48_2
ctr48_3
p48_2
p48_3
e
−4 0 4
control 24 h
poly(I:C) 24 h
control 48 h
poly(I:C) 48 h
ctr24_2
ctr24_3
ctr24_4
p24_2
p24_3
p24_4
ctr48_2
ctr48_3
ctr48_4
p48_2
p48_3
p48_4

Figure S3

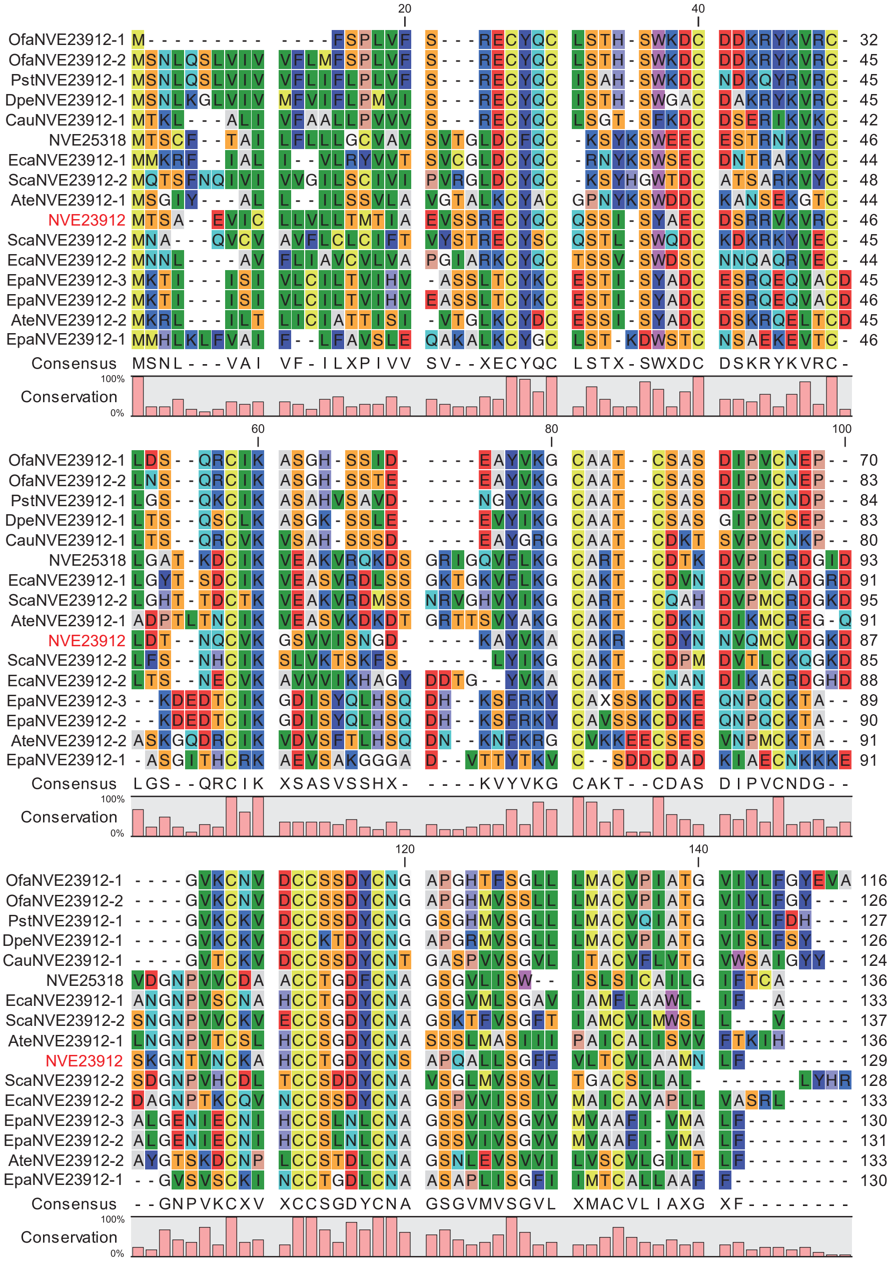
20
40
OfaNVE23912-1 M--------- ----FSPLVF S---RECYQC LSTH-SWKDC DDKRYKVRC- 32
OfaNVE23912-2 MSNLQSLVIV VFLMFSPLVF S---RECYQC LSTH-SWKDC DDKRYKVRC- 45
PstNVE23912-1 MSNLQSLVIV VFLIFLPLVF S---RECYQC ISAH-SWKDC NDKQYRVRC- 45
DpeNVE23912-1 MSNLKGLVIV MFVIFLPMVI S---RECYQC ISTH-SWGAC DAKRYKVRC- 45
CauNVE23912-1 MTKL---ALI VFAALLPVVV S---RECYQC LSGT-SFKDC DSERIKVKC- 42
NVE25318 MTSCF--TAI LFLLLGCVAV SVTGLDCFQC -KSYKSWEEC ESTRNKVFC- 46
EcaNVE23912-1 MMKRF--IAL I--VLRYVVT SVCGLDCYQC -RNYKSWSEC DNTRAKVYC- 44
ScaNVE23912-2 MQTSFNQIVI VVGILSCIVI PVRGLDCYQC -KSYHGWTDC ATSARKVYC- 48
AteNVE23912-1 MSGIY---AL L--ILSSVLA VGTALKCYAC GPNYKSWDDC KANSEKGTC- 44
NVE23912 MTSA--EVIC LLVLLTMTIA EVSSRECYQC QSSI-SYAEC DSRRVKVRC- 46
ScaNVE23912-2 MNA---QVCV AVFLCLCIFT VYSTRECYSC QSTL-SWQDC KDKRKYVEC- 45
EcaNVE23912-2 MNNL----AV FLIAVCVLVA PGIARKCYQC TSSV-SWDSC NNQAQRVEC- 44
EpaNVE23912-3 MKTI---ISI VLCILTVIHV -ASSLTCYKC ESTI-SYADC ESRQEQVACD 45
EpaNVE23912-2 MKTI---ISI VLCILTVIHV EASSLTCYKC ESTI-SYADC ESRQEQVACD 46
AteNVE23912-2 MKRL---ILT LICIATTISI -VTGLKCYDC ESSI-SYADC DSKRQELTCD 45
EpaNVE23912-1 MMHLKLFVAI F--LFAVSLE QAKALKCYGC LST-KDWSTC NSAEKEVTC- 46
Consensus MSNL---VAI VF-ILXPIVV SV--XECYQC LSTX-SWXDC DSKRYKVRC-
100%
Conservation
0%
60
80
100
OfaNVE23912-1 LDS--QRCIK ASGH-SSID- ----EAYVKG CAAT--CSAS DIPVCNEP-- 70
OfaNVE23912-2 LNS--QRCIK ASGH-SSTE- ----EAYVKG CAAT--CSAS DIPVCNEP-- 83
PstNVE23912-1 LGS--QKCIK ASAHVSAVD- ----NGYVKG CAAT--CSAS DIPVCNDP-- 84
DpeNVE23912-1 LTS--QSCLK ASGK-SSLE- ----EVYIKG CAAT--CSAS GIPVCSEP-- 83
CauNVE23912-1 LTS--QRCVK VSAH-SSSD- ----EAYGRG CAAT--CDKT SVPVCNKP-- 80
NVE25318 LGAT-KDCIK VEAKVRQKDS GRIGQVFLKG CART--CDTK DVPICRDGID 93
EcaNVE23912-1 LGYT-SDCIK VEASVRDLSS GKTGKVFLKG CAKT--CDVN DVPVCADGRD 91
ScaNVE23912-2 LGHT-TDCTK VEAKVRDMSS NRVGHVYIKG CART--CQAH DVPMCRDGKD 95
AteNVE23912-1 ADPTLTNCIK VEASVKDKDT GRTTSVYAKG CAKT--CDKN DIKMCREG-Q 91
NVE23912 LDT--NQCVK GSVVISNGD- ----KAYVKA CAKR--CDYN NVQMCVDGKD 87
ScaNVE23912-2 LFS--NHCIK SLVKTSKFS- -----LYIKG CAKT--CDPM DVTLCKQGKD 85
EcaNVE23912-2 LTS--NECVK AVVVIKHAGY DDTG--YVKA CAKT--CNAN DIKACRDGHD 88
EpaNVE23912-3 --KDEDTCIK GDISYQLHSQ DH--KSFRKY CAXSSKCDKE QNPQCKTA-- 89
EpaNVE23912-2 --KDEDTCIK GDISYQLHSQ DH--KSFRKY CAVSSKCDKE QNPQCKTA-- 90
AteNVE23912-2 ASKGQDRCIK VDVSFTLHSQ DN--KNFKRG CVKKEECSES VNPMCKTA-- 91
EpaNVE23912-1 -ASGITHCRK AEVSAKGGGA D--VTTYTKV C--SDDCDAD KIAECNKKKE 91
Consensus LGS--QRCIK XSASVSSHX- ----KVYVKG CAKT--CDAS DIPVCNDG--
100%
Conservation
0%
120
140
OfaNVE23912-1 ----GVKCNV DCCSSDYCNG APGHTFSGLL LMACVPIATG VIYLFGYEVA 116
OfaNVE23912-2 ----GVKCNV DCCSSDYCNG APGHMVSSLL LMACVPIATG VIYLFGY--- 126
PstNVE23912-1 ----GVKCKV DCCSSDYCNG GSGHMVSGLL LMACVQIATG IIYLFDH--- 127
DpeNVE23912-1 ----GVKCKV DCCKTDYCNG APGRMVSGLL LMACVPIATG VISLFSY--- 126
CauNVE23912-1 ----GVTCKV DCCSSDYCNT GASPVVSGVL ITACVFLVTG VWSAIGYY-- 124
NVE25318 VDGNPVVCDA ACCTGDFCNA GSGVLISW-- ISLSICAILG IFTCA----- 136
EcaNVE23912-1 ANGNPVSCNA HCCTGDYCNA GSGVMLSGAV IAMFLAAWL- IF--A----- 133
ScaNVE23912-2 SNGNPVVCKV ECCSGDYCNA GSKTFVSGFT IAMCVLMWSL L---V----- 137
AteNVE23912-1 LNGNPVTCSL HCCSGDYCNA SSSLMASIII PAICALISVV FTKIH----- 136
NVE23912 SKGNTVNCKA HCCTGDYCNS APQALLSGFF VLTCVLAAMN LF-------- 129
ScaNVE23912-2 SDGNPVHCDL TCCSDDYCNA VSGLMVSSVL TGACSLLAL- ------LYHR 128
EcaNVE23912-2 DAGNPTKCQV NCCSSDYCNA GSPVVISSIV MAICAVAPLL VASRL----- 133
EpaNVE23912-3 ALGENIECNI HCCSLNLCNA GSSVIVSGVV MVAAFI-VMA LF-------- 130
EpaNVE23912-2 ALGENIECNI HCCSLNLCNA GSSVIVSGVV MVAAFI-VMA LF-------- 131
AteNVE23912-2 AYGTSKDCNP LCCSTDLCNA GSNLEVSVVI LVSCVLGILT LF-------- 133
EpaNVE23912-1 --GVSVSCKI NCCTGDLCNA ASAPLISGFI IMTCALLAAF F--------- 130
Consensus --GNPVKCXV XCCSGDYCNA GSGVMVSGVL XMACVLIAXG XF--------
100%
Conservation
0%

Figure S4

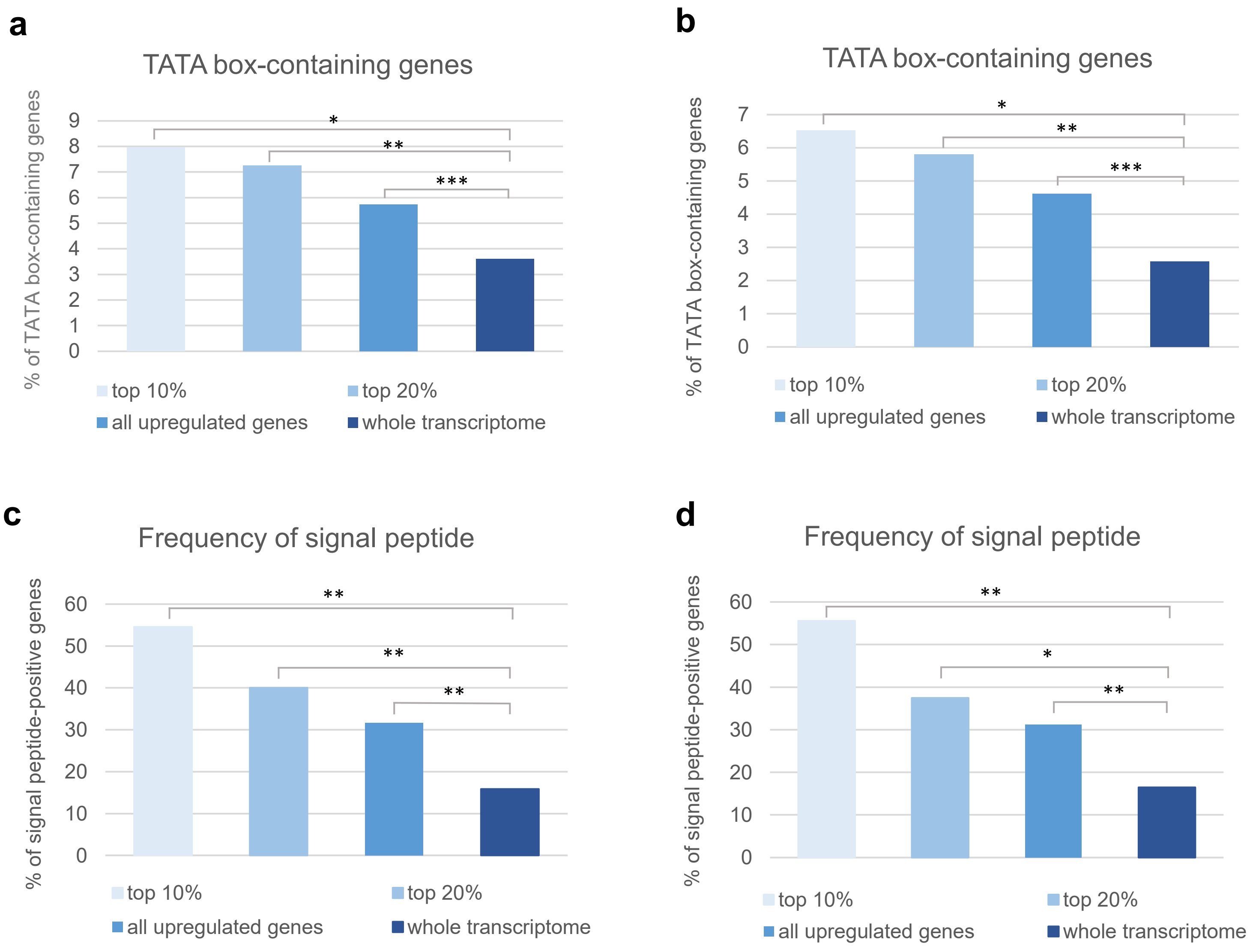
a
TATA box-containing genes
% of TATA box-containing genes
9
8
7
6
5
4
3
2
1
0
*
**
***
top 10%
top 20%
all upregulated genes
whole transcriptome
b
TATA box-containing genes
% of TATA box-containing genes
7
6
5
4
3
2
1
0
*
**
***
top 10%
top 20%
all upregulated genes
whole transcriptome
c
Frequency of signal peptide
% of signal peptide-positive genes
60
50
40
30
20
10
0
**
**
**
top 10%
top 20%
all upregulated genes
whole transcriptome
d
Frequency of signal peptide
% of signal peptide-positive genes
60
50
40
30
20
10
0
**
*
**
top 10%
top 20%
all upregulated genes
whole transcriptome

Figure S5

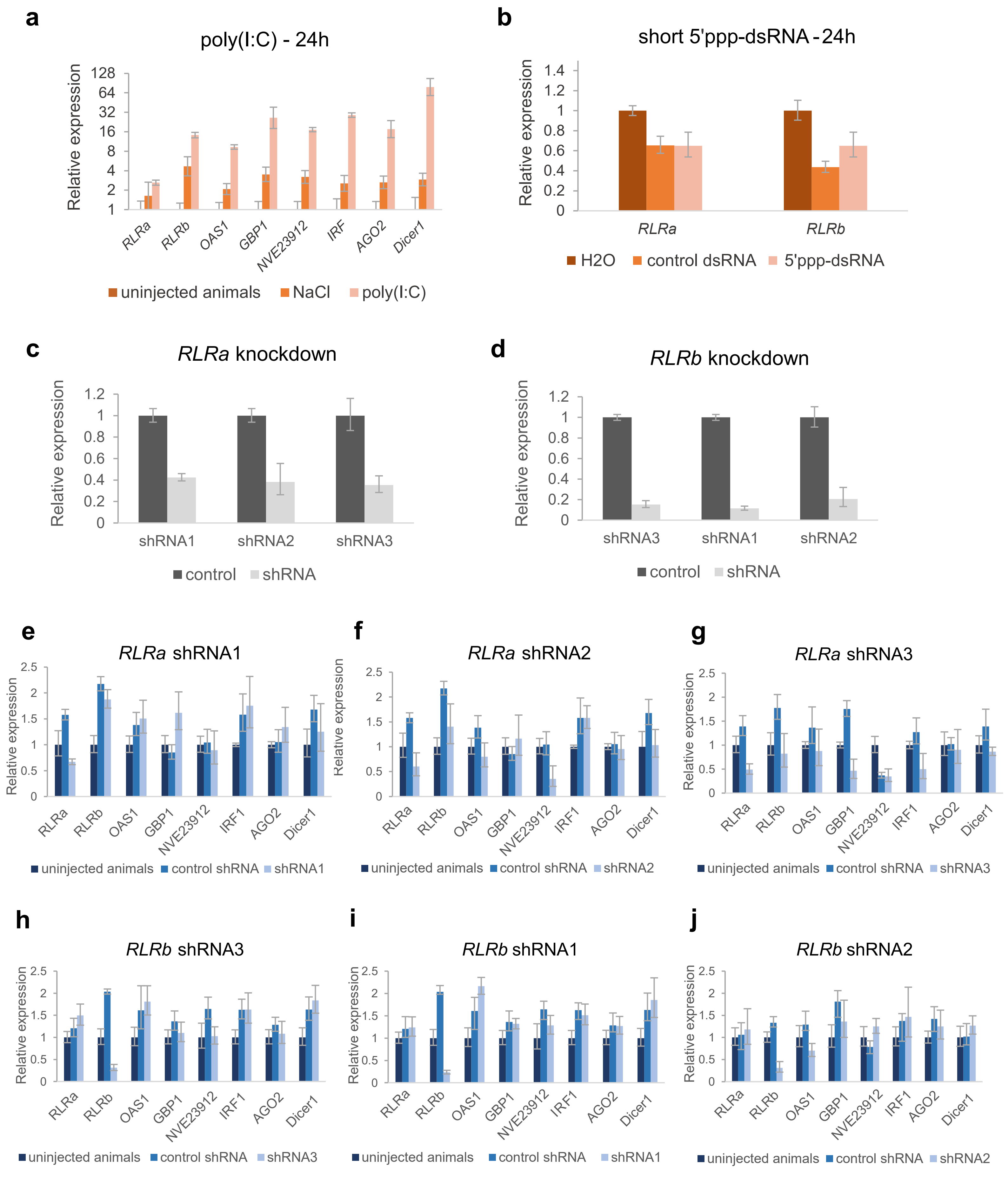
a
poly(I:C) - 24h
Relative expression
128
64
32
16
8
4
2
1
RLRa
RLRb
OAS1
GBP1
NVE23912
IRF
AGO2
Dicer1
uninjected animals
NaCl
poly(I:C)
b
short 5'ppp-dsRNA - 24h
Relative expression
1.4
1.2
1
0.8
0.6
0.4
0.2
0
RLRa
RLRb
H2O
control dsRNA
5'ppp-dsRNA
c
RLRa knockdown
Relative expression
shRNA1
shRNA2
shRNA3
control
shRNA
d
RLRb knockdown
Relative expression
shRNA3
shRNA1
shRNA2
control
shRNA
e
RLRa shRNA1
f
RLRa shRNA2
g
RLRa shRNA3
h
RLRb shRNA3
i
RLRb shRNA1
j
RLRb shRNA2
Relative expression
RLRa
RLRb
OAS1
GBP1
NVE23912
IRF1
AGO2
Dicer1
uninjected animals
control shRNA
shRNA1
shRNA2
shRNA3

Figure S6

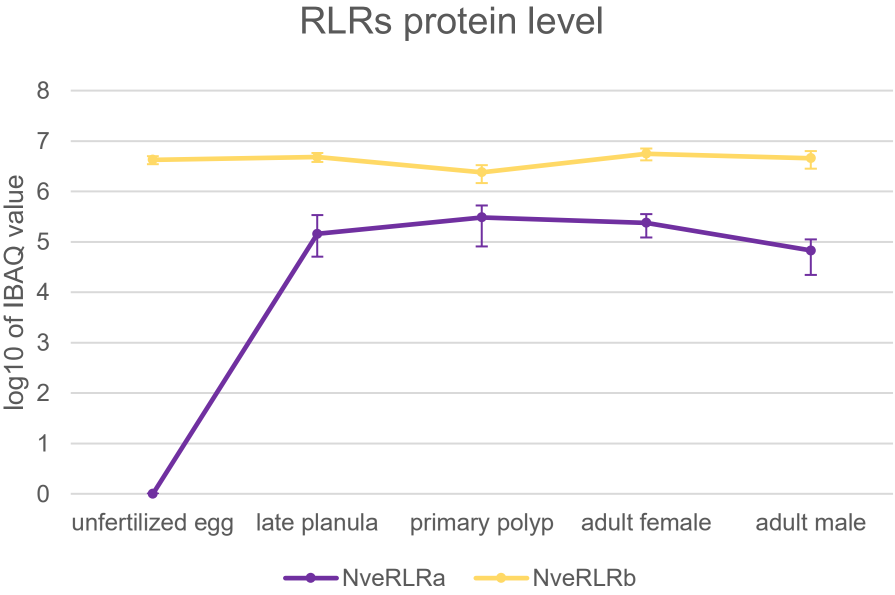

RLRs protein level
log10 of IBAQ value
8
7
6
5
4
3
2
1
0
unfertilized egg
late planula
primary polyp
adult female
adult male
NveRLRa
NveRLRb

Figure S7

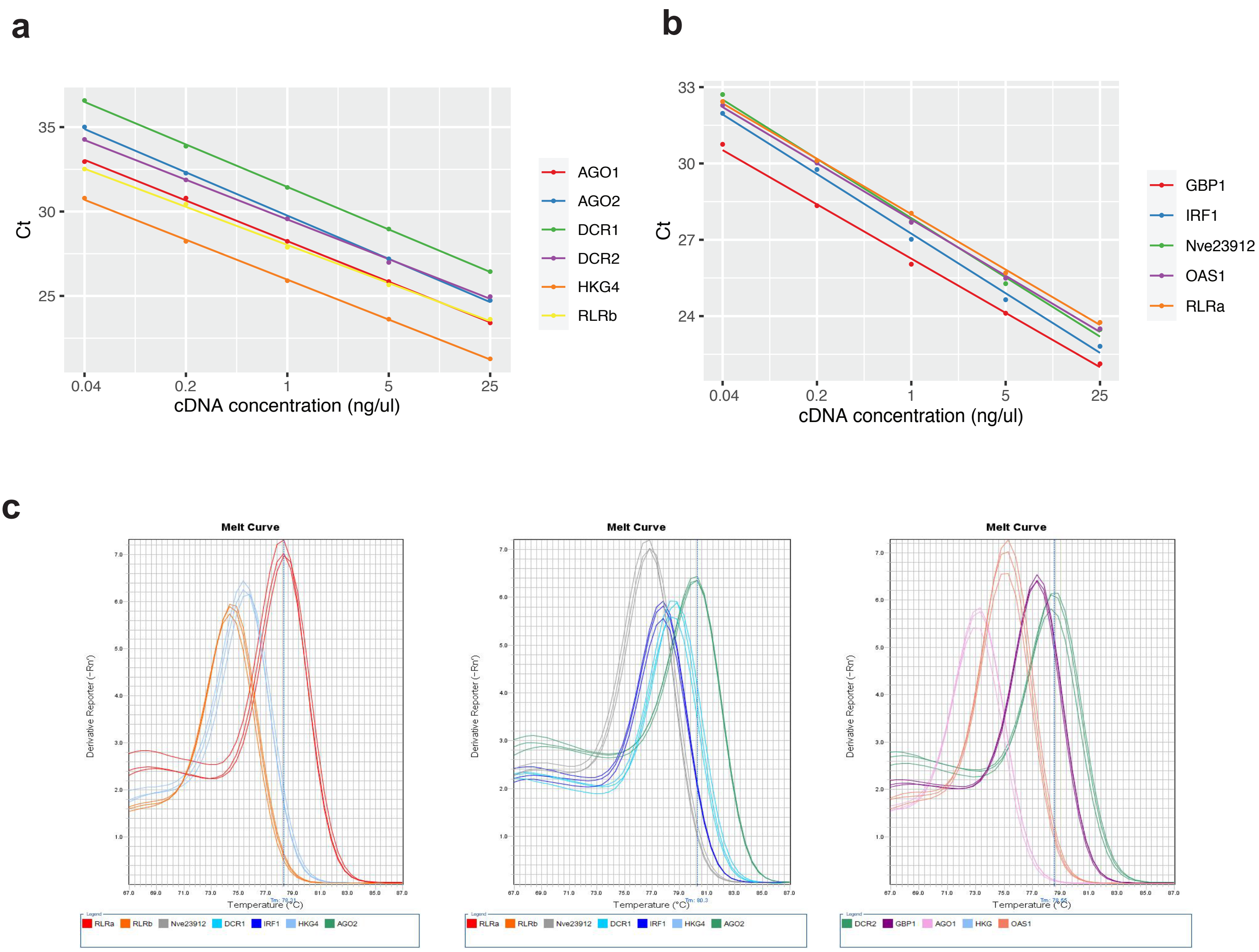
a
Ct
35
30
25
0.04
0.2
1
5
25
cDNA concentration (ng/ul)
AGO1
AGO2
DCR1
DCR2
HKG4
RLRb
b
Ct
33
30
27
24
0.04
0.2
1
5
25
cDNA concentration (ng/ul)
GBP1
IRF1
Nve23912
OAS1
RLRa
c
Melt Curve
Derivative Reporter (−Rn')
Temperature (°C)
RLRa RLRb Nve23912 DCR1 IRF1 HKG4 AGO2
Melt Curve
Derivative Reporter (−Rn')
Temperature (°C)
RLRa RLRb Nve23912 DCR1 IRF1 HKG4 AGO2
Melt Curve
Derivative Reporter (−Rn')
Temperature (°C)
DCR2 GBP1 AGO1 HKG OAS1
